# Multi-trait GWAS of reaction norm parameters reveals environment-responsive loci influencing reproductive performance in heifers

**DOI:** 10.1186/s40104-026-01428-5

**Published:** 2026-06-08

**Authors:** Lucio F. M. Mota, Leonardo M. Arikawa, Daniel J. A. Santos, Luiz F. Brito, Larissa F. S. Fonseca, Henrique N. Oliveira, Lucia G. Albuquerque

**Affiliations:** 1 Department of Animal Science, São Paulo State University (UNESP), School of Agricultural and Veterinarian Sciences, Jaboticabal, SP 14884-900 Brazil; 2 Department of Animal Sciences, Purdue University, West Lafayette, IN 47907 USA; 3 National Council for Science and Technological Development, Brasilia, DF 71605-001 Brazil

**Keywords:** Bayesian fine-mapping, Endocrine–metabolic integration, Environmental responsiveness, Reaction norm, Reproductive plasticity

## Abstract

**Background:**

Reproductive efficiency in Nellore heifers is fundamental to the profitability and sustainability of beef production in tropical regions, where environmental stress can cause genotype-environment (G×E) interactions that affect fertility. Using 200,258 and 299,885 phenotypic records for heifer early pregnancy (HP) and heifer rebreeding (HR), respectively, we investigated the genetic basis of reproductive plasticity via single-step genomic reaction norms across a continuous environmental gradient (EG) defined from yearling weight records as a proxy for environmental quality. Genomic analyses included 22,556 animals (21,456 females and 1,100 sires) with genotypes imputed to 409,617 single-nucleotide polymorphisms (SNPs). We then performed genome-wide association analyses of the reaction norm intercept (genetic merit) and slope (environmental sensitivity), followed by multi-trait summary analyses and Bayesian fine-mapping of significant loci using imputed whole-genome sequence variants within ±100 kb windows around lead SNPs.

**Results:**

Heritability for both traits increased with environmental quality, indicating environment-dependent expression of genetic variance. Genetic correlations for HP and HR across the environmental gradient ranged from 0.15 to 0.98, supporting substantial G×E interactions and reranking between low and high environmental conditions. Multi-trait analyses of reaction norm parameters identified 482 significant signals for the intercept and 700 for the slope. Intercept-associated loci were enriched for lipid metabolism, embryonic development, estrous regulation, and hypothalamic–pituitary signaling, whereas slope-associated loci highlighted endocrine signaling, metabolic plasticity, and neuroendocrine feedback responsive to contrasting post-weaning nutritional and management conditions captured by the EG. Fine-mapping refined associations to 146 (intercept) and 149 (slope) putative loci for HP, and 117 (intercept) and 167 (slope) for HR, supported by high posterior probabilities and Bayes factors. Candidate variants mapped to endocrine and metabolic regulators, including *IGF1*, *LEP*, *GHRL*, *GNRHR*, *KISS1*, *MAPK3*, *PLAG1*, *INSR*, and *LHCGR*.

**Conclusions:**

G × E interactions play a key role in shaping the genetic architecture of reproductive efficiency in Nellore heifers. Integrating reaction norm, multi-trait GWAS, and fine-mapping highlighted loci, affecting both genetic merit and environmental sensitivity of fertility, providing targets to select more resilient Nellore females for tropical systems.

**Graphical Abstract:**

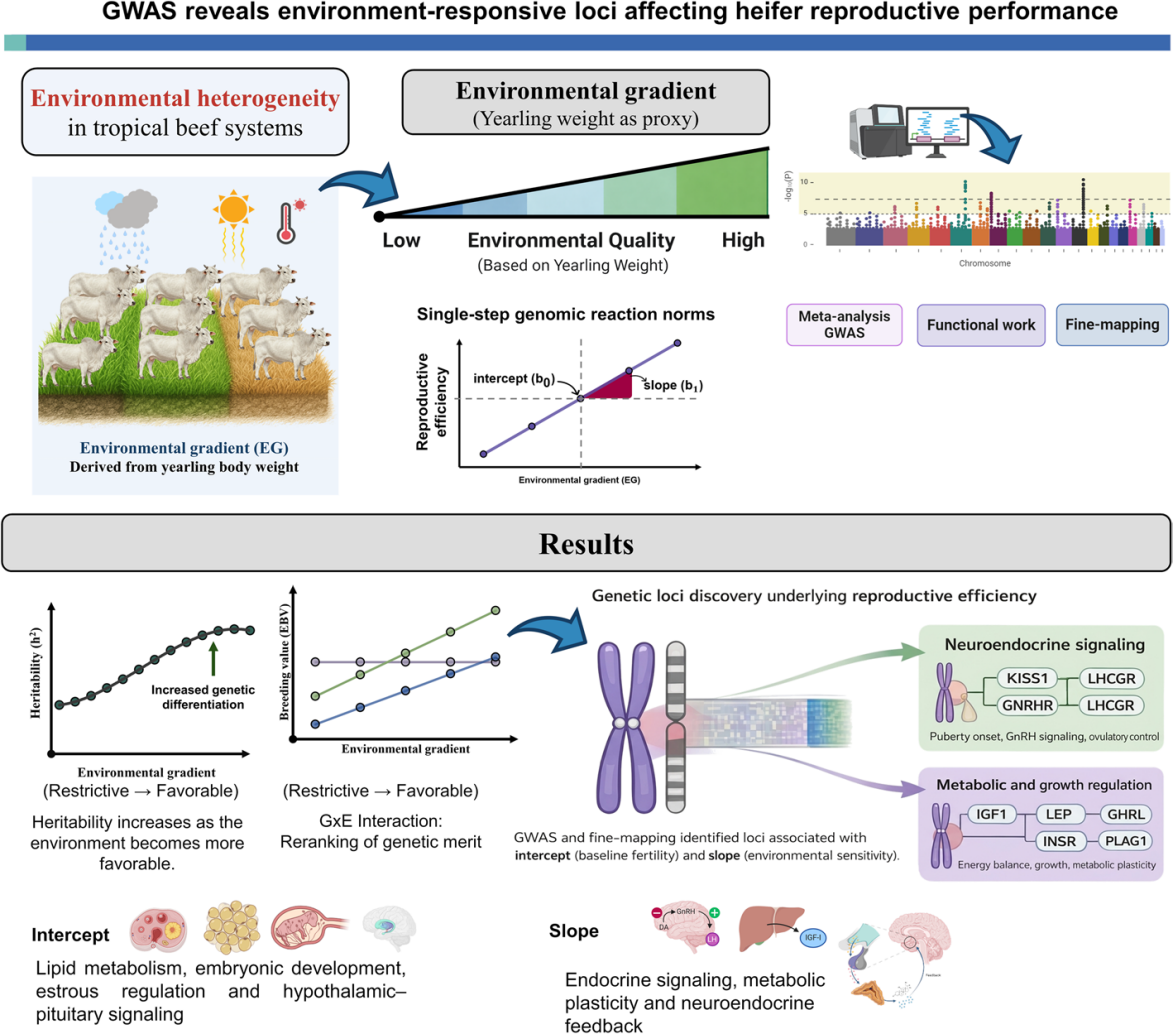

**Supplementary Information:**

The online version contains supplementary material available at 10.1186/s40104-026-01428-5.

## Background

Reproductive performance in heifers is a key determinant of productivity and overall sustainability in beef cattle systems, with early pregnancy and successful rebreeding being critical to maximizing the lifetime efficiency of heifers [1]. These traits directly influence the number of younger heifers exposed to reproduction and the number of calves weaned during their productive life [1, 2]. However, reproductive efficiency, as assessed by heifer early pregnancy (HP) and heifer rebreeding (HR), is strongly affected by environmental factors such as heat stress and inadequate nutrition [3–5]. These stressors can disrupt the neuroendocrine regulation of the reproductive axis, impairing the hormonal control of puberty onset, cyclicity, and conception [6, 7]. Nutritional status is particularly important, as it is closely associated with both HP and HR, because energy metabolism plays a major role in regulating essential reproductive hormones [8, 9]. Consequently, low HP and HR rates can result in long-term productivity losses that are difficult to reverse. Therefore, selecting replacement heifers with better reproductive performance potential is crucial for the sustainability of beef cattle production systems [1].

Nellore heifers (*Bos taurus indicus*) in tropical production systems are predominantly raised on pasture and exposed to a wide range of harsh environmental conditions, facing challenges such as seasonal poor nutrition, high temperatures, and humidity. These stressor factors often lead to genotype-by-environment (G×E) interactions [10, 11], which represent a substantial source of genetic variation in reproductive traits and can affect the ranking of animals and reduce genetic progress [12, 13]. For instance, heifers that perform well in a favorable environment may exhibit reduced reproductive performance under stressful conditions, such as high heat or low nutrient availability [11, 14]. Given the complexity of reproductive efficiency traits, which have a complex inheritance controlled by multiple genomic regions [3, 15, 16], their expression is often modulated by environmental factors [5, 17]. Environmental stressors, such as nutritional deficits or heat stress, may hinder the major metabolic and endocrine pathways that regulate the onset of puberty [16, 18, 19]. The hypothalamic–pituitary–gonadal axis, which controls the reproductive system, is highly sensitive to changes in the external environment and nutritional aspects affecting yearling weight, which can affect sexual maturity and fertility. As a result, G×E interactions may cause notable changes in the genomic regions affecting reproductive traits, depending on the environmental conditions [17].

The dynamic relationship between genetics and environment complicates the development of breeding programs aimed at improving reproductive efficiency [5]. Thus, a better understanding of these biological processes is essential for developing effective selection strategies based on reproductive performance. Identifying genomic regions that contribute to resilience and high reproductive efficiency across environments is challenging due to the many physiological and environmental factors involved. Breeders are interested in animals that can maintain reproductive performance despite environmental stressors. These animals have genetic variants that help them handle heat stress, poor nutrition, and other challenges without significant declines in reproductive function. Selecting for greater resilience is a long-term strategy to improve the adaptability of cattle herds to climate change and fluctuating environmental conditions [20].

Genome-wide association studies (GWAS) have been successfully used in livestock to identify genetic variants associated with several reproductive traits that cause genetic differences among animals [21–25]. When GWAS is integrated with reaction norm (RN) models, it can enable the identification of genomic regions whose effects on reproductive traits also depend on environmental conditions [8, 17, 26]. RN models are particularly useful for studying G×E interactions because they describe how an individual’s performance changes across a range of environmental conditions [27]. Usually, reproductive efficiency indicators in heifers, such as HP and HR, are strongly influenced by G×E interactions [5, 28–30]. Combining results from single-trait GWAS for the intercept and slope of the RN can enhance the ability to identify shared genomic variants across different environmental gradient (EG) levels and reveal possible pleiotropic effects on different reproductive traits [21, 31, 32]. Hence, the main objective of this study was to identify genomic regions affecting RN model parameters (intercept and slope) and to explore the biological mechanisms of candidate genes involved in the reproductive performance of Nellore heifers.

## Methods

### Field data

Reproductive data were recorded on 379,228 Nellore heifers born between 1987 and 2021 in 455 farms across all five regions of Brazil, Paraguay, Colombia, and Bolivia. These data are part of three major commercial breeding programs (DeltaGen; Cia do Melhoramento and Paint), which are integrated into the Nellore Alliance dataset (www.gensys.com.br).

Reproductive performance was assessed based on heifer early pregnancy (HP) and heifer rebreeding (HR), as described below. The dataset exhibits a high degree of genetic connectedness across herds, due to the widespread use of artificial insemination (AI), which accounted for over 62% of recorded births. Its large scale and wide-ranging environmental and management conditions make this dataset an excellent resource for evaluating the sensitivity of reproductive performance in heifers to environmental variation.

The animal handling procedure used followed ARRIVE (Animal Research: Reporting of In Vivo Experiments) guidelines and was approved by the Ethical Committee of the São Paulo State University (Jaboticabal, SP, Brazil; protocol number: 007757/18).

### Phenotypic information

The HP trait was defined by assigning a value of 1 (success) to heifers that first calved until 28 months and 0 (failure) for those that calved later. HR was defined by attributing 1 (success) to heifers that had a second calf and 0 (failure) to heifers that did not have a second calf in the next calving season. The yearling weight (YW, in kg) was measured in heifers at an average age of 514 ± 63.41 d.

Contemporary groups (CG) for all traits were defined by animals born in the same year and season, raised on the same farm (at birth, weaning, and yearling), and managed under similar conditions throughout these stages. For HR, the effects of previous pregnancy status (early or regular season, as described below) and first calf sex were also included in the CG definition. Including early or regular season code in the CG accounts for herd-specific breeding schemes, where two reproductive seasons were implemented for heifers: 1) an anticipated breeding season (March–April) lasting 60 d, and 2) a regular breeding season (November–January) lasting 90 d. Compared to the regular season, heifers bred in the anticipated season typically had more time to recover body weight following first calving before their first rebreeding, potentially influencing HR rate [1]. In CG, calf sex was considered to adjust for its physiological effects on dam reproductive performance, since male calves increase gestational energy needs and may extend postpartum recovery, potentially decreasing the chances of timely rebreeding [33, 34].

Phenotypic quality control (QC) for HP and HR excluded CGs where all animals exhibited the same binary response (0 or 1), thus lacking variability. For $\mathrm{YW}$, phenotypic data exceeding ±3.5 standard deviations from the CG mean were removed. Additionally, CGs with fewer than nine records were excluded for all traits (HP, HR and YW). Following quality control, 200,258 records remained for HP, with 57% of heifers classified as precocious (age at first calving ≤ 28 months), and 299,885 records were retained for HR, with 63% of cows with a second calf. The HP and HR probability distribution across herd are shown in Additional file 2: Fig. S1.

### Genomic data

A total of 21,456 females were genotyped with commercial SNP panels of different marker density: Illumina BovineHD 770K (*n* = 1,259 individuals), 50K (*n* = 11,490), GGP 75K (*n* = 1,370), Zip Deoxys 27K (*n* = 4,166), and 20K (*n* = 3,171) and 1,100 sires were genotyped with Illumina BovineHD (770K, Illumina Inc., San Diego, CA, USA). Markers located on non-autosomal chromosomes or with duplicate genomic coordinates were removed from subsequent analyses. A pre-imputation QC filter also removed SNPs with a GenCall score < 0.80, to prevent genotyping errors.

Low- and medium-density genotypes were imputed to high density using the FImpute v3 software [35] based on a reference population of 6,862 Nellore animals. The HD reference Nellore animals belonged to the same breeding population as the target animals (i.e., those genotyped with low- and medium-density SNP panels) and were selected to maximize connectedness with the target population. This strategy was designed to capture the main haplotypes segregating in the population, including data from key sires and highly genetically connected cows (see Additional file 2: Fig. S2). Imputation accuracy was assessed by splitting the reference population into five folds and masking markers to mimic low- and medium-density arrays, resulting in an allelic correlation of 0.97 [36]. Population structure was assessed by principal component analysis (PCA) using the *ade4* R package [37]. The PCA scatter plot (first versus second PCAs) confirmed the genetic relationship between genotyped sires and females, with the absence of population substructure (see Additional file 2: Fig. S2). Post-imputation QC excluded SNPs with minor allele frequency (MAF) < 0.05, Hardy–Weinberg equilibrium *P*-value < 10^−6^, and call rate < 0.90 for both SNPs and samples. Thus, the final dataset included 22,556 animals and 409,617 SNPs for further analyses.

### Statistical modeling

The dataset used to evaluate reproductive sensitivity to environmental variation belonged to commercial herds with diverse management practices and climatic conditions, allowing for a thorough assessment of reproductive performance sensitivity to environmental factors. These herds were distributed in different geographical regions of Brazil, Bolivia, Colombia, and Paraguay, with annual rainfall varying from ~700 to ~3,000 mm and dry seasons lasting up to seven months in some regions.

Genetic sensitivity to environmental variation was evaluated using a two-step approach [5]. Firstly, the environmental gradient (EG) values were derived from CG solutions based on Best Linear Unbiased Estimates (BLUE) using YW as the phenotype. The BLUE solutions served as an indicator of EG quality, as it reflects nutritional conditions and post-weaning management​​, a critical phase for metabolism and reproductive development [14]. YW was chosen due to its biological connection to metabolic status and puberty attainment in beef heifers, where variations in production systems and environments that influence YW significantly impact the early sexual puberty of heifers [1, 18].

The EG was obtained using an animal model based on the single-step GBLUP (ssGBLUP) approach as follows:

$${\boldsymbol{y}}={\boldsymbol{X}}{\boldsymbol{b}}+{\boldsymbol{Z}}{\boldsymbol{a}}+{\boldsymbol{e}},$$

where ${\boldsymbol{y}}$ is the vector of phenotypic data for YW; ${\boldsymbol{b}}$ represents the fixed effect of CG and age as a linear covariate; $\boldsymbol{a}$ is the vector of additive genetic effect, assumed to follow a normal distribution $N(0,{\boldsymbol{H}}{\sigma }_{a}^{2})$ where ${\sigma }_{a}^{2}$ is the additive genetic variance, and ${\boldsymbol{H}}$ is the pedigree-genomic relationship matrix. The residual effect (${\boldsymbol{e}}$) was assumed to follow $N(0,{\boldsymbol{I}}{\sigma }_{e}^{2})$ with ${\sigma }_{e}^{2}$ being the residual variance and ${\boldsymbol{I}}$ an identity matrix. ${\boldsymbol{X}}$ and ${\boldsymbol{Z}}$ are the incidence matrices linking phenotypic records to fixed (${\boldsymbol{b}}$) and additive genetic (${\boldsymbol{a}}$) effects, respectively.

In the ssGBLUP model, the inverse of $${\boldsymbol{H}}$$$$({{\boldsymbol{H}}}^{-1})$$ was calculated as [38]: $${{\boldsymbol{H}}}^{-1} ={\boldsymbol{ }{\boldsymbol{A}}}^{-1}+\left[\begin{array}{cc}0& 0\\ 0& {{\boldsymbol{G}}}^{-1}- {{\boldsymbol{A}}}_{22}^{-1}\end{array}\right]$$ where $${{\boldsymbol{A}}}^{-1}$$ represents the pedigree relationship matrix for all animals included in the pedigree, $${{\boldsymbol{A}}}_{22}^{-1}$$ represents the inverse of the pedigree-based relationship matrix for the genotyped animals, and $${{\boldsymbol{G}}}^{-1}$$ is the inverse of the genomic-based relationship matrix. The ***G*** matrix [39] was calculated as: $$\boldsymbol{G}=\frac{{\boldsymbol{M}}{{\boldsymbol{M}}}^{\prime}}{2\sum_{j=1}^{m}{p}_{j}\left(1-{p}_{j}\right)}$$ where $${\boldsymbol{M}}$$ is the SNP marker matrix (coded as 0, 1, and 2 for genotypes AA, AB, and BB) adjusted for allele frequency ($$2{p}_{j}$$), and $${p}_{j}$$ is the MAF. The CG solutions describing the EG were standardized (mean = 0 and variance = 1), with values ranging from −3.0 to +3.0. The analyses were conducted using the blupf90 + program [40].

### Reaction norm (RN) model

After standardizing the BLUE solutions of the YW GC effects, the intercept and linear covariates for different EG values were calculated using a Legendre function. Thus, a single-step genomic RN (ssGRN) model was used to evaluate the G×E interaction as follows:

$$\boldsymbol{l} = \boldsymbol{Xb} + \boldsymbol{Z}_{\boldsymbol{int}}\boldsymbol{a}_{\boldsymbol{int}} + \boldsymbol{Z}_{\boldsymbol{slop}}\boldsymbol{a}_{\boldsymbol{slop}} + \boldsymbol{e}$$

where ${\boldsymbol{l}}$ is a vector of underlying liability for HP and HR, ${\boldsymbol{b}}$ is the vector of the fixed effect of CG, ${\boldsymbol{X}}$ is the incidence matrix connecting ***b*** to ${\boldsymbol{l}}$. The incidence matrices ${{\boldsymbol{Z}}}_{{\boldsymbol{i}}{\boldsymbol{n}}{\boldsymbol{t}}}$ and ${{\boldsymbol{Z}}}_{{\boldsymbol{s}}{\boldsymbol{l}}{\boldsymbol{o}}{\boldsymbol{p}}}$, with values based on Legendre orthogonal polynomial functions of the EG levels, link the random additive genetic effects for the intercept (${{\boldsymbol{a}}}_{{\boldsymbol{i}}{\boldsymbol{n}}{\boldsymbol{t}}}$**)** and slope (${{\boldsymbol{a}}}_{{\boldsymbol{s}}{\boldsymbol{l}}{\boldsymbol{o}}{\boldsymbol{p}}}$) to ${\boldsymbol{l}}$, respectively, and $\boldsymbol{e}$ is the residual effect. The model was fitted based on a threshold ssGRN model and assuming the distribution: $f(y|{\iota }_{i})= \prod_{i=1}^{{n}_{i}}1\left({\iota }_{i}<{t}_{i}\right)1\left(y=0\right)+1\left({\iota }_{i}>{t}_{i}\right)1(y=1),$ where *y* is the binary trait (HP or HR), ${\iota }_{i}$ is the liability for observation *i*, ${t}_{i}$ is the threshold for binary response, and ${n}_{i}$ is the number of information.

The additive genetic and residual effects were considered normally distributed: ${\boldsymbol{a}}=\left\{{a}_{j}\right\}\sim N\left(0, {\boldsymbol{H}}\otimes {\boldsymbol{K}}\right)$ and ${\boldsymbol{e}}=\left\{{e}_{ij}\right\}\sim N(0, {\boldsymbol{I}}{\sigma }_{e}^{2}),$ where $${\boldsymbol{K}}=\left[\begin{array}{cc}{\sigma }_{{a}_{int}}^{2}& {\sigma }_{{a}_{int},{a}_{slop}}\\ {\sigma }_{{a}_{int},{a}_{slop}}& {\sigma }_{{a}_{slop}}^{2}\end{array}\right]$$ is the genetic variance–covariance matrix for the intercept (${{\boldsymbol{a}}}_{{\boldsymbol{i}}{\boldsymbol{n}}{\boldsymbol{t}}}$) and slope (${{\boldsymbol{a}}}_{{\boldsymbol{s}}{\boldsymbol{l}}{\boldsymbol{o}}{\boldsymbol{p}}}$) of the ssGRN model and ⊗ denotes the Kronecker product.

The analyses were conducted using the gibbsf90 + program [40]. The Gibbs sampler consisted of a chain of 500,000 cycles, with the first 100,000 iterations discarded as burn-in and samples stored every ten cycles, resulting in 40,000 posterior samples used to estimate genetic parameters. Convergence was assessed by visual inspection using the Bayesian Output Analysis (BOA) R package [32] and confirmed with the Geweke test [41] (*P*-value > 0.25 for HP and *P*-value > 0.13 for HR).

### Genetic parameters

Marginal posterior distributions for additive genetic variance $\left({\widehat{\sigma }}_{aE{G}_{j}}^{2}\right)$ and heritability $\left({\widehat{h}}_{E{G}_{j}}^{2}\right)$ for HP and HR across the *j*^th^ EG levels were obtained from the retained posterior samples. For each posterior sample, additive genetic variance for HP and HR across the EG levels were calculated based on the equations: ${\widehat{\sigma }}_{a{EG}_{j}}^{2}= {\Phi }_{f}{\boldsymbol{k}}{\Phi }_{f'}$; where ${\Phi }_{f'}$ is the Legendre orthogonal polynomial estimate (intercept and slope) corresponding to each EG level and ***K*** is the additive genetic (co)variance matrix for the intercept and slope. The heritability (${\widehat{h}}_{{EG}_{j}}^{2}$) for each EG level (*j*) was determined as follows: ${\widehat{h}}_{{EG}_{j}}^{2}=\frac{{\widehat{\sigma }}_{a{EG}_{j}}^{2}}{{\widehat{\sigma }}_{a{EG}_{j}}^{2}+{\widehat{\sigma }}_{e{EG}_{j}}^{2}}$; ${\widehat{\sigma }}_{a{EG}_{j}}^{2}$ is the additive genetic variance and ${\widehat{\sigma }}_{e{\mathrm{EG}}_{j}}^{2}$ is the residual variance at EG level *j*. The genetic correlation across EG levels ($r_{\mathrm{EG}_{j},\mathrm{EG}_{j}\prime}$) was determined as: ${r}_{EGj, EGj\prime}={\sigma }_{EGj,EGj\prime}/\sqrt{{\widehat{\sigma }}_{a{EG}_{j}}^{2}*{\widehat{\sigma }}_{a{EG}_{j\prime}}^{2}}$, where ${\upsigma }_{\mathrm{EG}_{j},\mathrm{EG}_{j}\prime}$ represents the covariance between EG level *j* and EG level *j*', estimated in the same way as the additive genetic variance for each EG level. To summarize the results, we extracted genetic correlations among three target EG levels: Low (−3.0 SD), Medium (0.0 SD), and High (3.0 SD), and across all available EG levels.

The predicted genomic estimated breeding values (GEBV – ${\widehat{\mathrm{g}}}_{{\mathrm{EG}}_{ij}}$) for each animal *i* at a given EG level *j* were obtained as: ${\widehat{\mathrm{g}}}_{{\mathrm{EG}}_{ij}}= {{\mathrm{a}}_{i}\Phi }_{{\mathrm{f}}_{j}}\prime$, where ${a}_{i}$ is the vector of estimated (G)EBV for the intercept (${\boldsymbol{a}}_{\mathbf{i}\mathbf{n}\mathbf{t}}$) and slope (${\boldsymbol{a}}_{\mathbf{s}\mathbf{l}\mathbf{o}\mathbf{p}}$) of animal *i* and ${\Phi }_{{\mathrm{f}}_{j}}\prime$ is the transpose vector of the Legendre orthogonal polynomial for the *j* EG level. To assess and visualize the magnitude of G × E for reproductive traits, RN analyses were performed for the 90 sires with the highest GEBV at medium EG (EG = 0) and largest numbers of phenotyped progeny (mean = 150 ± 110; range = 21 to 2,910), across low (EG < −2.5), medium (−0.2 < EG < 0.2), and high (EG > 2.5) EG (Fig. 3a and c).

### Detection of candidate genomic regions

To identify genomic regions associated with these RN parameters for HP and HR, GWAS results were summarized separately for the intercept and slope using a multi-trait Chi-square statistic based on signed *t*-values. This procedure follows the general multi-trait summary-statistic framework proposed by Bolormaa et al. [32] based on signed *t*-values. For each RN parameter $p$ (intercept or slope) and each trait $q$ (HP or HR), SNP effects ${\widehat{{\boldsymbol{u}}}}_{{\boldsymbol{p}},{\boldsymbol{q}}}$ were obtained by back-solving the GEBV of genotyped animals from the equation $\hat{\boldsymbol{u}}_{\boldsymbol{p,q}} = \boldsymbol{M}^{\prime} [\boldsymbol{M}\boldsymbol{M}^{\prime}]^{-\boldsymbol{1}}\hat{\boldsymbol{a}}_{\boldsymbol{p,q}}$, where ${\boldsymbol{M}}$ is the centered genotype matrix and ${\widehat{{\boldsymbol{a}}}}_{{\boldsymbol{p}},{\boldsymbol{q}}}$ is the vector of GEBV for the corresponding RN parameter for reproductive trait.

Signed *t*-values were computed as [9]: ${t}_{k,p,q}=\frac{{\widehat{u}}_{k,p,q}}{se({\widehat{u}}_{k,p,q})}$ where ${t}_{k,p,q}$ is the *t*-value for the SNP marker *k* of RN parameter *p* and *q* trait; ${\widehat{u}}_{k,p,q}$ is the SNP effects for the RN coefficient (intercept or slope) on HR and HP traits and $sd({\widehat{u}}_{k,p,q})$ is the standard deviation for the SNP effect.

Multi-trait SNP association test was applied separately for the intercept and slope using a Chi-square (${\upchi }^{2}$) test with two degrees of freedom, summarizing signed *t*-values from HP and HR. For each SNP marker (*n* = 409,617), the statistic was: ${\chi }_{k,p}^{2}={T}_{k,p}^{\prime}{V}_{p}^{-1}{T}_{k,p}$, where ${T}_{k,p}=[{t}_{k,p,HP},{t}_{k,p,HR}{]}^{\prime}$ is the vector of signed *t*-values for HP and HR for RN parameter $p$, and ${V}_{p}$ is the across-SNP correlation matrix of signed *t*-values between HP and HR for the same RN parameter. Thus, the multi-trait statistic combined information across traits within a given RN parameter, whereas intercept and slope were evaluated in separate analyses.

Genomic inflation was evaluated using the λ factor, calculated as $\uplambda ={}^{\text{median }({\upchi }^{2})}\!\left/ \!{}_{0.456}\right.$. The λ values ranged from 1.0 to 1.1 in this study, which are considered acceptable values [42]. To balance false-positive control and statistical power, we adopted a trait-wise, linkage disequilibrium (LD)-adjusted multiple-testing threshold based on the effective number of independent chromosomal segments (${M}_{e}$), rather than on the total number of SNPs. Because LD reduces the number of effectively independent marker tests, the total SNP count may overestimate the true multiple-testing burden, particularly in cattle populations with extended LD. For this reason, LD-adjusted thresholds based on the effective number of independent tests have been used in livestock GWAS [43, 44]. The Me was estimated as a function of effective population size (Ne) and genome length (L, in Morgans) for the SNPs considered in this study (24.77 M; see Additional file 1: Table S1), following Goddard et al. [45]: $\mathrm{Me}=\left(2\times{Ne}\times{L}\right)/\log\left(Ne\times{L}\right)$, where log() represents the natural logarithm. The GWAS significance threshold was $P<\alpha /{M}_{e}$, we consider a $\alpha =0.01$ with an adjusted *P*-value of 9.91e^−6^ (0.01/1,105.55) with a ${-\mathrm{log}}_{10}\left(P\mathrm{-value}\right)=5.04\sim5.0$.

To assess the predicted SNP effect across the EG level, we selected the SNP markers that exhibited a $-\mathrm{log}_{10}\left(P\mathrm{-value}\right)> 5$ for both, intercept and slope. For each significant SNP (*k*), the predicted SNP effect (${\widehat{\mathrm{w}}}_{kj}$) for HP and HR across environmental gradients (*j*) was computed using a linear prediction equation: ${\widehat{w}}_{kj}={\boldsymbol{u}_{k}\Phi }_{{\mathrm{f}}_{j}}\prime$ where ${\boldsymbol{u}}_{k}$ is the vector of SNP-specific RN (intercept and slope) [5, 46].

### Gene mapping of significant SNP for GWAS statistical summary

SNP markers from the GWAS summary were deemed significant when –log_10_(*P*-value) > 5.0 based on Me Bonferroni correction test. Genes located within a ± 100 kb genomic window of each SNP were annotated using the *Bos taurus* ARS-UCD1.3 genome assembly, via the BioMart R package [47]. Functional enrichment for biological processes (Gene Ontology, GO) and pathways was performed using clusterProfiler [48], with the terms tested via hypergeometric distribution and FDR-adjusted *P*-value < 0.05.

### Fine-mapping of significant genomic regions

To prioritize candidate variants underlying the meta-GWAS signals for RN parameters, we performed Bayesian fine-mapping using the FINEMAP-adj framework [49], which uses LMM-derived association statistics and a relatedness-adjusted LD matrix for samples of related individuals. Prior to fine-mapping, genotyped animals for 409,617 SNPs were imputed to the whole-genome sequence (WGS) level using the FImpute v3 software [35], restricting the imputation target to ±100 kb windows surrounding the significant SNPs detected in the multi-trait GWAS statistical combination for the intercept and slope parameters. The WGS reference panel comprised 243 sequenced Nellore sires, including 52 sires sequenced with Illumina HiSeq X™ Ten and 191 sires with Illumina NovaSeq™, from the same breeding population as the target animals. This reference population represents an updated version of the reference population described by Fernandes Júnior et al. [50], with the key sires selected by k-means clustering of the genomic relationship matrix, with the sire showing the largest number of genotyped progeny chosen for sequencing within each cluster. The sequencing procedures are described in Fernandes Júnior et al. [50]. The genomic PCA showed overlap between sequenced sires and genotyped animals, supporting the representativeness of the reference panel (see Additional file 2: Fig. S3).

Within each locus-specific window, variant-level quality control retained WGS variants with MAF > 0.05, call rate > 0.95, and no extreme departure from Hardy–Weinberg equilibrium (*P*-value > 1 × 10⁻⁶). GWAS summary statistics for intercept and slope of reproductive performance were obtained using mixed linear model-based association (MLMA) approach under a leave-one-chromosome-out (LOCO) strategy in GCTA v. 1.95 [51]. Fine-mapping was then conducted using the FINEMAP-adj framework described by Wang et al. [49], with adapted inputs designed for related individuals (LD and MLMA results). This strategy was adopted to control individual relatedness and extended LD, in Nellore cattle.

Statistical support for variant prioritization was summarized using posterior inclusion probabilities (PIP) and log_10_ (Bayes factor). PIP denotes the probability that a variant belongs to the causal set at a locus, obtained by summing the posterior probabilities of all causal models that include that variant. In FINEMAP-adj, the Bayes factor measures the evidence supporting inclusion of a variant in the causal set based on the GWAS summary statistics and the relatedness-adjusted LD matrix using a shotgun stochastic search (SSS). Variants with PIP > 0.90 and ${\mathrm{log}}_{10}(Bayes factor)> 3.5$ were considered high-priority candidate variants. Fine-mapping results were generated separately for RN intercept (genetic merit) and RN slope (environmental responsiveness) and are reported as locus-level PIP landscapes (see Additional file 2: Figs. S7 and S9) and as top prioritized variants.

## Results

### Genetic parameters

#### Heritability estimates

**Fig. 1:**
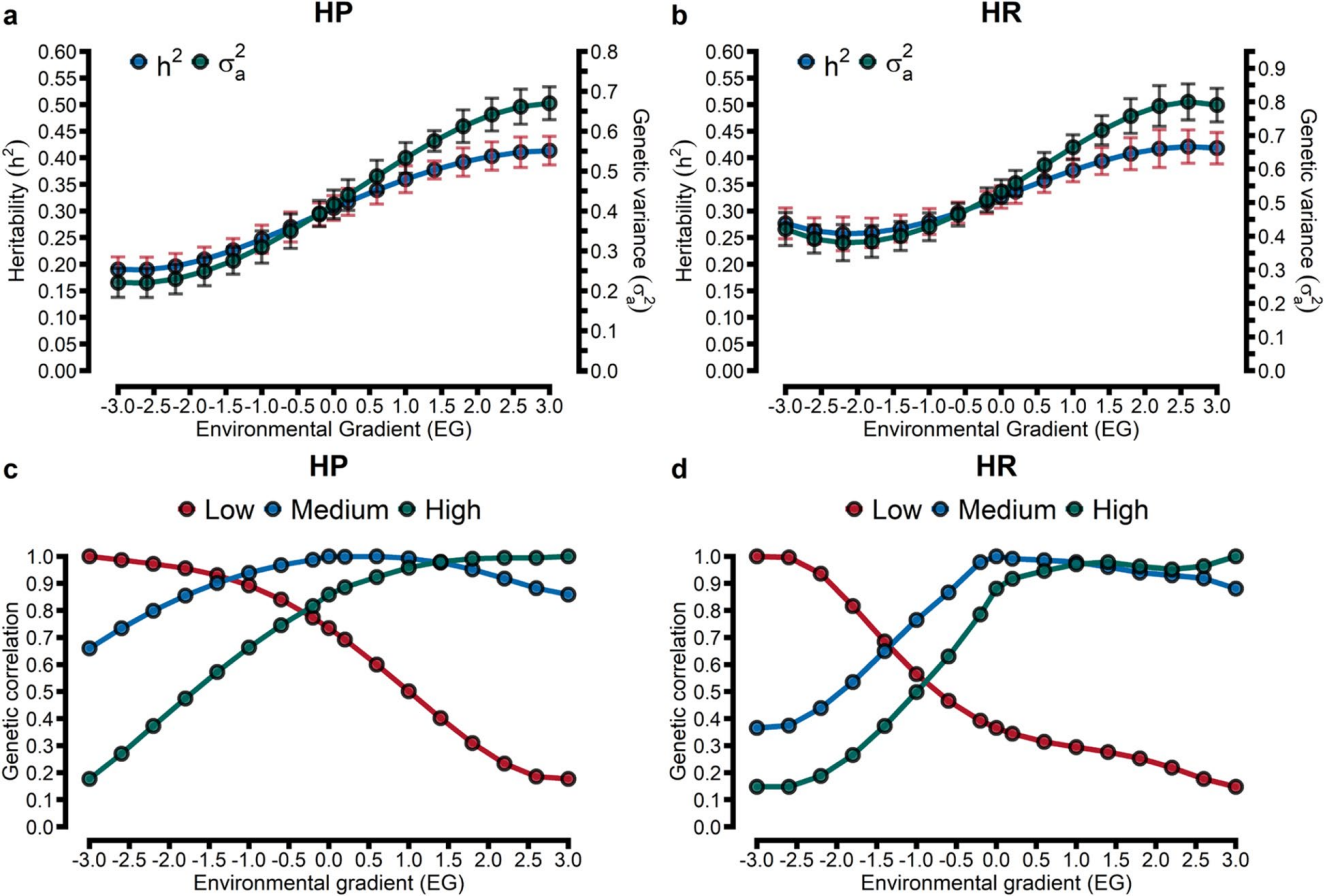
Environmental dependency of genetic variance, heritability, and genetic correlation estimates for reproductive traits in Nellore heifers. Heritability (*h*^2^, blue lines) and additive genetic variance (σ^2^ₐ, green lines) for heifer early pregnancy (HP, **a**) and heifer rebreeding (HR, **b**) across the environmental gradients (EG). Genetic correlations for HP (**c**) and HR (**d**) across EG, comparing low (EG = −3.0; red), medium (EG = 0.0; blue), and high (EG = 3.0; green) EG levels

Heritability estimates for HP and HR, varied markedly across the EG levels, providing clear evidence of G×E interaction (Fig. 1a and b). Under unfavorable EG conditions (EG = −3.0), heritability was 0.19 ± 0.02 for HP and 0.26 ± 0.03 for HR. As environmental conditions improved, heritability increased progressively and nonlinearly, reaching 0.41 ± 0.03 for HP and 0.42 ± 0.03 for HR under the most favorable EG (EG = 3.0). Across intermediate levels (−2.5 to 2.5), heritability estimates increased gradually, indicating that environmental improvement enhances both genetic and phenotypic expression. Notably, HP exhibited a pronounced sigmoidal response across the EG levels became more favorable compared to HR (Fig. 1a and b). This difference was accompanied by proportional increases in additive genetic variance for HP (204.3%) than for HR (87.7%), whereas phenotypic variance increased more moderately (HP: 24.3%; HR: 37.8%). The steeper increase in genetic variance relative to phenotypic variance under favorable EG explains the marked increase in heritability, for HP (117.6%) and HR (51%). These patterns demonstrate that environmental improvement preferentially enhances the expression of additive genetic variance, reinforcing the central role of EG when evaluating fertility-related traits. The contrasting variance trajectories between HP and HR further suggest trait-specific genetic architectures and distinct modes of G × E modulation, with direct implications for breeding strategy design.

#### Genetic correlation across environments

To characterize how environmental heterogeneity reshapes the genetic architecture of HP and HR, we quantified genetic correlations (*r*_g_​) between EG levels. Both traits showed a clear decay in genetic correlations as the EG difference increased (Fig. 1c and d). For HP, *r*_g_ ranged from 0.18 between extreme EGs (EG: −3.0 vs. 3.0) to 0.99. When contrasting low EG with other levels *r*_g_ averaged 0.63 ± 0.29 (Fig. 1c), whereas comparisons between medium EG remained consistently high varying from 0.66 to 0.99 (mean = 0.90 ± 0.10). Correlations involving high EG were intermediate (mean = 0.73 ± 0.28), ranging from 0.18 to 0.99. These results suggest a stable genetic performance for HP under medium EG but marked G × E when animals are under extreme EG levels (Fig. 1c).

For HR, G × E effects were more pronounced (Fig. 1d), with genetic correlations varying from 0.15 to 0.98 (mean = 0.45 ± 0.27) between lower and other EG levels. Medium-to-other comparisons ranged from 0.37 to 0.99 (mean = 0.78 ± 0.23) and high-to-other EG comparisons from 0.14 to 0.98 (mean = 0.66 ± 0.34; Fig. 1d). In both traits, *r*_g_ dropped below the conventional 0.80 threshold as environmental contrast increased, confirming biologically relevant G × E and highlighting greater environmental susceptibility for HR compared to HP.

#### Reaction norm parameters reflect partially overlapping genetic architectures

**Fig. 2:**
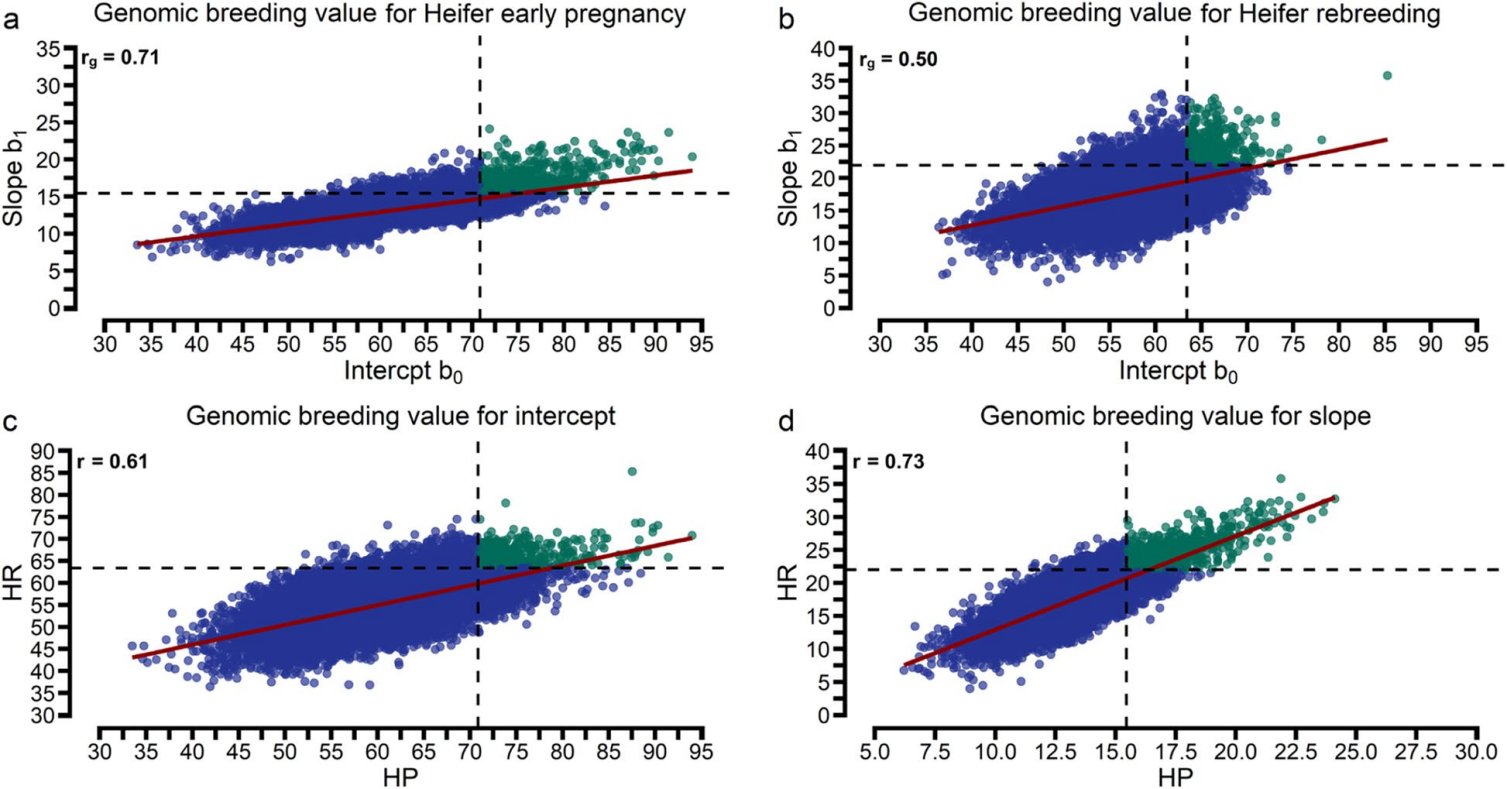
Genetic correlation estimates and genomic estimated breeding values (GEBVs) for reaction norm parameters of reproductive traits in Nellore heifers. Genetic correlations between intercept (b_0_) and slope (b_1_) of the reaction norm model for heifer early pregnancy (HP, **a**) and heifer rebreeding (HR, **b**), capturing the relationship between baseline reproductive potential and environmental sensitivity. Pearson correlations between GEBVs of HP and HR for intercept (**c**) and slope (**d**)

Reaction norm (RN) modeling provided insight into the genetic association between reproductive potential and environmental sensitivity. The genetic correlation between the intercept (b_0_​) and slope (b_1_​) of the RN was high for HP (*r*_*g*_ = 0.71; 95% HPD [0.71, 0.72]) and moderate for HR (*r*_*g*_​ = 0.50; 95% HPD [0.49, 0.51]), indicating partially coordinated but non-identical genetic regulation of performance and plasticity (Fig. 2a and b). At the GEBV level, Pearson correlations between HP and HR were moderate for the intercept (*r* = 0.61; 95% credible interval [0.59, 0.61]; Fig. 2c), but higher slope (*r* = 0.73; 95% HPD [0.72, 0.73]; Fig. 2d). Suggesting more similar genetic basis for plastic responses among HP and HR. This supports the hypothesis of shared regulatory mechanisms for genetic potential and reproductive adaptation in response to environmental improvements.

#### *Sire-specific G* × *E highlighting variable reproductive plasticity*

**Fig. 3:**
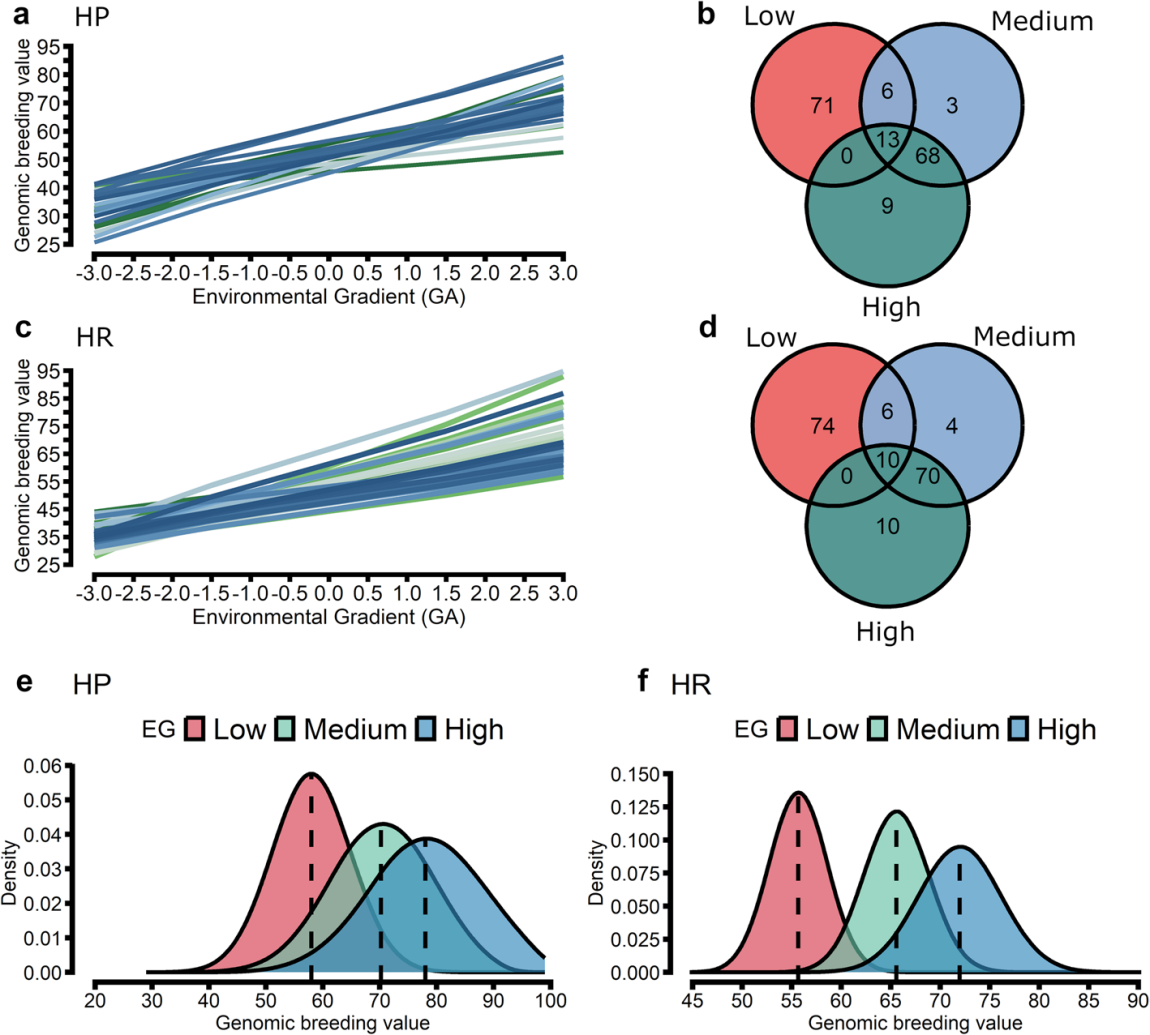
Reaction norm trajectories and sire consistency across environmental gradients for reproductive traits in Nellore heifers. Reaction norm trajectories of genomic estimated breeding values (GEBVs) for heifer early pregnancy (HP, **a**) and heifer rebreeding (HR, **c**) across environmental gradient (EG) levels. Each line represents one of the top 90 sires selected based on GEBV at medium EG (EG = 0.0), illustrating variability in genetic responsiveness across low (EG = −3.0), medium (EG = 0.0), and high (EG = 3.0) environmental conditions. Venn diagrams showing the overlap among the top 90 ranked sires selected at low, medium and high EG for HP (**b**) and HR (**d**). Density plots showing the distribution of GEBVs for sires across low, medium, and high EG for HP (**e**) and HR (**f**)

Substantial sire-level heterogeneity in environmental responsiveness was observed for both HP and HR, as evidenced by different GEBV trajectories across EG levels (Fig. 3a and c). RN for the top 90 sires (mean progeny = 150 ± 110; range = 21 to 2,910) demonstrated marked variability in both magnitude and direction (Fig. 3a and c), consistent with the moderate-to-high intercept–slope correlations (Fig. 2a and b).

Although most sires showed increasing GEBVs under favorable environments, the extent of this response varied considerably, revealing distinct plasticity profiles (Fig. 3a and c). At the population level, mean GEBV increased from 58% to 78% for HP and from 55% to 72% for HR between the lowest and highest EG levels (Fig. 3e and f). These shifts were accompanied by changes in genetic variance and GEBV distributions, illustrating how environmental context reshapes both performance and selection potential (Fig. 3). This environmental sensitivity translated into substantial re-ranking of sires across EG conditions (Fig. 3b and d). Venn diagram analyses showed that only 13 sires for HP and 10 for HR remained among the top performers across low, medium and high EG classes (Fig. 3b and d). In contrast, overlap was substantially higher between medium and high EG, with 68 common sires for HP and 70 for HR, indicating context-dependent robustness of genetic merit.

### Genomic mapping of reaction norm effects for reproductive traits

#### SNP effects across environmental gradients

**Fig. 4:**
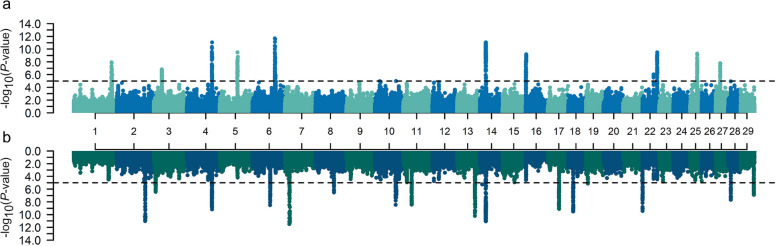
Miami plot from the GWAS statistical combination of reaction norm parameters in reproductive traits. Significant SNP associations for the intercept (**a**) and slope (**b**) parameter of the reaction norm model. The dashed line denotes the significance threshold ($-\!\log_{10}(P\mathrm{-value})>5$)

Multi-trait meta-GWAS of reaction norm parameters identified extensive genomic control of both reproductive potential (intercept) and environmental responsiveness (slope). A total of 482 and 700 SNPs were associated with intercept and slope, respectively (Fig. 4). Among these, 131 SNPs were shared by RN parameters and mainly located on BTA4 (92.38–93.81 Mb) and BTA14 (23.03–24.06 Mb). These genomic regions harbor genes involved in the hypothalamic–pituitary–ovarian axis (Tables 1, 2 and 3), supporting the observed correlations between RN parameters (Fig. 2a and b).

**Table 1:** Candidate genes mapped on genomic regions deemed significant for the intercept of reaction norm affecting Nellore heifer's early pregnancy and heifer rebreeding

BTA	Windows region^a^	n (SNP)^b^	HP ${\sigma }_{a}^{2}, \%$	HR ${\sigma }_{a}^{2}, \%$	−log_10_(*P*-value)^c^	Genes
1	138.67–138.80	7	1.50–2.81	0.98–1.41	5.17–7.94	*NEK11, U6, ASTE1, ATP2C1*
1	139.23–139.39	3	1.00–1.16	1.11–4.34	5.41–6.99	*PSMG1, BRWD1, GET1, SH3BGR, LCA5L*
1	139.76–139.92	8	1.48–1.48	0.97–4.14	5.61–7.82	*B3GALT5, IGSF5, PCP4*
1	140.17–140.61	4	1.17–1.27	1.06–4.13	5.02–6.86	*DSCAM, U6*
1	141.39–141.40	2	0.96–3.00	1.11–4.45	5.98–5.98	*BACE2*
3	29.14–29.39	26	0.15–0.25	0.95–1.33	5.09–6.83	*SYT6, OLFML3, HIPK1, DCLRE1B, AP4B1*
3	29.49–29.88	28	0.35–0.55	0.98–1.48	5.04–6.67	*OLFML3, HIPK1, DCLRE1B, AP4B1, BCL2L15, PTPN22, RSBN1, PHTF1, MAGI3*
4	92.38–93.11	40	1.09–3.87	0.95–1.57	5.21–11.05	*SND1, MIR129–1, LEP, RBM28, PRRT4, IMPDH1, HILPDA, GARIN1A, GARIN1B, CALU, OPN1SW, CCDC136, FLNC, ATP6V1F, SPMIP1, KCP, IRF5, TNPO3, TSPAN33, SMO*
4	93.23–93.81	7	1.01–1.93	0.98–2.44	5.70–9.18	*SMO, AHCYL2, NRF1, MIR183, UBE2H, ZC3HC1, KLHDC10*
5	65.32–65.69	11	1.04–2.16	0.96–2.29	5.43–8.12	*UTP20, ARL1, SPIC, MYBPC1, CHPT1, SYCP3, GNPTAB, DRAM1*
5	65.97–66.23	20	0.95–1.56	0.97–2.26	5.14–10.00	*WASHC3, NUP37, PARPBP, PMCH, IGF1, U6*
5	66.44–66.79	6	1.12–2.05	0.95–1.55	5.04–8.09	*U6, PAH, ASCL1, U1*
6	83.15–83.49	19	1.27–2.98	1.06–1.82	5.3–11.89	*CENPC, STAP1, UBA6, GNRHR, TMPRSS11D*
6	84.86–85.17	6	0.96–2.86	0.99–1.55	5.42–5.92	*MGC152010, UGT2A1, SULT1B1*
14	23.03–24.06	84	1.08–4.39	0.96–4.50	5.03–11.05	*XKR4, TMEM68, TGS1, LYN, RPS20, U1, MOS, PLAG1, CHCHD7, SDR16C5, SDR16C6, PENK, U6, BPNT2*
16	0.81–1.31	17	1.04–2.64	0.98–2.57	5.02–8.04	*TMEM183A, PPFIA4, MYOG, U6, ADORA1, MYBPH, CHI3L1, FMOD, PRELP, OPTC*

^a^ Significant SNP markers were grouped into windows with a maximum gap of 200 kb between markers

^b^ The number of significant markers

^c^ The minimum and maximum significant values

**Table 2:** Candidate genes mapped on genomic regions deemed significant for the intercept of reaction norm affecting heifers' early pregnancy and heifer rebreeding in Nellore cattle heifers

BTA	Windows region^a^	n (SNP)^b^	HP${\sigma }_{a}^{2}, \%$	HR${\sigma }_{a}^{2}, \%$	–log_10_(*P*-value)^c^	Genes
16	1.45–1.50	2	0.99–3.29	0.97–1.91	6.93–7.89	*ATP2B4, LAX1, ZBED6*
16	1.70–2.01	31	1.59–2.62	0.98–2.05	5.58–9.19	*ZBED6, SNRPE, SOX13, ETNK2, REN, KISS1, GOLT1A, PLEKHA6*
22	41.12–41.29	5	1.04–2.16	1.04–1.67	5.53–6.04	*FHIT*
22	54.26–54.75	52	0.96–2.45	0.95–3.52	5.09–9.11	*CLEC3B, EXOSC7, ZDHHC3, TMEM42, GHRL, SEC13, ATP2B2, bta–mir–885*
22	54.86–55.11	8	1.01–3.05	0.99–1.28	5.04–9.49	*SLC6A1, HRH1*
25	25.25–25.28	2	0.95–1.04	0.98–1.56	5.45–5.7	*KATNIP, GSG1L*
25	25.68–25.72	2	0.96–1.45	0.98–1.78	5.18–5.41	*GSG1L, XPO6, SBK1*
25	25.83–25.93	4	1.11–1.51	0.99–1.15	5.02–5.42	*SBK1, LAT, SPNS1, NFATC2IP, CD19, RABEP2, ATP2A1*
25	25.93–26.43	19	1.09–1.98	0.97–1.49	5.26–9.3	*LAT, SPNS1, NFATC2IP, CD19, RABEP2, ATP2A1, SH2B1, TUFM, ATXN2L, EIF3CL, CLN3, IL27, SGF29, NUPR1, SULT1A1, SLX1A, CORO1A, MAPK3, GDPD3, YPEL3, TBX6, PPP4C, ALDOA, bta–mir–12060, TLCD3B, C16orf92, DOC2A, INO80E, HIRIP3, TAOK2, TMEM219, KCTD13, ASPHD1, SEZ6L2, CDIPT, MVP, bta–mir–2325c, PAGR1, MAZ, bta–mir–2385, KIF22, ZG16, U6, C25H16orf54, QPRT, SPN*
25	26.66–26.92	16	0.99–1.29	0.99–1.15	6.07–9.11	*MYL11, SEPTIN1, ZNF48, ZNF771, DCTPP1, ITGAL, ZNF688, ZNF689, PRR14, FBRS, SRCAP, SNORA30, TMEM265, U6, PHKG2, CFAP119, RNF40, ZNF629, BCL7C, CTF1*
27	16.22–16.61	19	0.99–2.36	0.95–2.39	5.03–8.08	*TLR3, FAM149A, CYP4V2, KLKB1, F11, MTNR1A, FAT1*

^a^ Significant SNP markers were grouped into windows with a maximum gap of 200 kb between markers

^b^ Number of significant markers

^c^ minimum and maximum significant value

**Table 3:** Candidate genes mapped on genomic regions deemed significant for the slope of reaction norm affecting Nellore heifers' early pregnancy and heifer rebreeding

BTA	Windows region^a^	n (SNP)^b^	HP ${\sigma }_{a}^{2}, \%$	HR ${\sigma }_{a}^{2}, \%$	–log_10_(*P*-value)^c^	Genes
2	104.62–105.61	32	1.09–2.29	1.01–2.75	5.58–11.16	*IGFBP2, IGFBP5*
3	5.95–6.15	8	2.25–2.45	0.95–2.20	5.07–5.35	*NUF2, RGS5*
3	6.29–6.30	3	1.50–2.02	1.05–1.82	5.29–5.32	*RGS5, RGS4*
3	6.61–6.67	8	0.96–1.22	1.75–2.91	5.28–6.43	*CCDC190, HSD17B7, DDR2*
3	7.06–7.09	2	1.10–1.18	2.83–3.33	5.1–5.33	*UHMK1, SH2D1B, NOS1AP, SPATA46*
3	7.44–7.57	7	1.22–1.56	3.02–3.53	5.13–5.69	*NOS1AP, SNORA70, OLFML2B, ATF6*
4	92.28–93.38	116	1.23–2.19	1.17–3.04	5.04–9.17	*SND1, LRRC4, MIR129–1, LEP, RBM28, PRRT4, IMPDH1, HILPDA, GARIN1A, GARIN1B, CALU, OPN1SW, CCDC136, FLNC, bta–mir–2422, ATP6V1F, SPMIP1, KCP, IRF5, TNPO3, bta–mir–1843, TSPAN33, SMO, AHCYL2, STRIP2, NRF1*
4	93.75–93.81	5	0.95–1.18	0.98–1.22	5.14–7.38	*UBE2H, ZC3HC1, KLHDC10*
6	64.93–65.28	14	1.01–1.66	1.22–3.46	5.09–8.52	*GABRA2, COX7B2*
6	65.45–65.91	22	0.97–1.26	1.01–3.11	5.14–7.71	*COX7B2, GABRA4, GABRB1, GABRA2, U6*
7	16.09–16.19	7	3.15–3.62	2.28–3.47	5.13–11.75	*INSR, ARHGEF18*
7	16.96–17.02	7	1.08–2.85	1.04–3.64	5.44–10.24	*CERS4, CD320, NDUFA7, RPS28, KANK3, ANGPTL4, RAB11B, MARCHF2, HNRNPM, PRAM1, ZNF414, MYO1F*
7	17.19–17.25	5	1.35–2.66	1.22–2.86	6.61–10.24	*HNRNPM, PRAM1, ZNF414, MYO1F, SNORA70, ADAMTS10, NFILZ, ACTL9, OR2Z1*
7	17.37–17.75	18	2.41–2.92	0.98–2.75	5.03–11.55	*U7, VAV1, SH2D3A, TRIP10, C3, TNFSF14*
7	17.98–18.02	7	1.09–2.29	1.01–2.75	10.1–11.42	*CD70, TNFSF9, TUBB4A, DENND1C, CRB3, SLC25A23, SLC25A41, KHSRP, GTF2F1, ALKBH7, CLPP, ACER1*
8	68.48–68.66	13	0.95–2.61	0.97–2.10	5.07–6.51	*–*
10	76.38–76.47	28	1.28–2.58	2.10–3.01	5.19–8.87	*ESR2, U6, MTHFD1*
10	78.57–78.60	4	1.07–1.25	0.97–1.27	5.05–7.30	*CCDC196, GPHN*
10	78.95–79.19	9	2.25–3.59	1.28–2.05	6.08–7.07	*GPHN, GARIN2, PALS1, ATP6V1D, EIF2S1, PLEK2*
11	30.98–31.69	56	1.07–2.65	1.01–2.64	5.08–8.82	*STON1, GTF2A1L, LHCGR, FSHR*
13	65.68–65.97	18	1.35–1.69	1.66–2.87	5.21–8.37	*DLGAP4, MYL9, TGIF2, RAB5IF, SLA2, NDRG3, DSN1, MTCL2, SAMHD1, TLDC2, RBL1*
13	66.18–66.25	11	1.51–2.03	0.98–1.85	6.13–10.80	*MROH8, RPN2, GHRH, MANBAL, SRC*
13	66.45–66.45	2	0.98–1.00	1.01–1.12	5.42–5.86	*BLCAP, NNAT*
13	66.62–66.73	4	1.07–1.12	1.18–1.61	5.25–7.09	*CTNNBL1, VSTM2L*
13	67.03–67.23	9	1.15–1.48	1.58–1.86	5.03–9.43	*RPRD1B, TGM2, U6, KIAA1755, BPI, LBP, SNORA71*
14	10.38–10.38	1	0.98	1.01	5.24–5.24	*ASAP1*
14	23.03–24.06	84	1.01–3.28	1.05–5.34	5.03–11.75	*XKR4, TMEM68, TGS1, LYN, RPS20, U1, MOS, PLAG1, CHCHD7, SDR16C5, SDR16C6, PENK, U6, BPNT2*
17	41.11–41.98	62	1.00–1.17	0.95–2.49	5.08–9.10	*GRIA2, bta–mir–2321, GLRB*
18	18.29–18.87	45	0.11–0.30	0.37–0.53	5.06–9.75	*ZNF423, CNEP1R1, HEATR3, TENT4B, ADCY7, BRD7, NKD1*
19	6.84–6.84	1	0.42	0.46	5.13–5.13	*ANKFN1*
22	0.22–0.38	19	1.12–2.40	1.55–2.79	5.06–9.83	*MRPS24, URGCP, UBE2D4, DBNL, PGAM2*
22	0.51–0.61	6	0.97–1.15	1.04–1.60	5.81–7.05	*VOPP1*
22	0.81–0.96	16	1.00–1.80	2.88–3.81	5.57–9.35	*LANCL2, EGFR*
28	10.10–10.39	15	1.02–3.50	1.02–3.54	5.28–7.87	*RYR2*
29	48.90–49.49	23	0.97–4.07	1.11–1.73	5.21–6.90	*KCNQ1, TRPM5, TSSC4, TSPAN32, ASCL2, TH, INS, IGF2, MRPL23, TNNT3*
29	49.76–49.94	5	1.15–3.55	0.98–1.08	5.23–6.68	*CTSD, IFITM10, DUSP8, MOB2*

^a^ Significant SNP markers were grouped into windows with a maximum gap of 200 kb between markers

^b^ Number of significant markers

^c^ minimum and maximum significant value

**Fig. 5:**
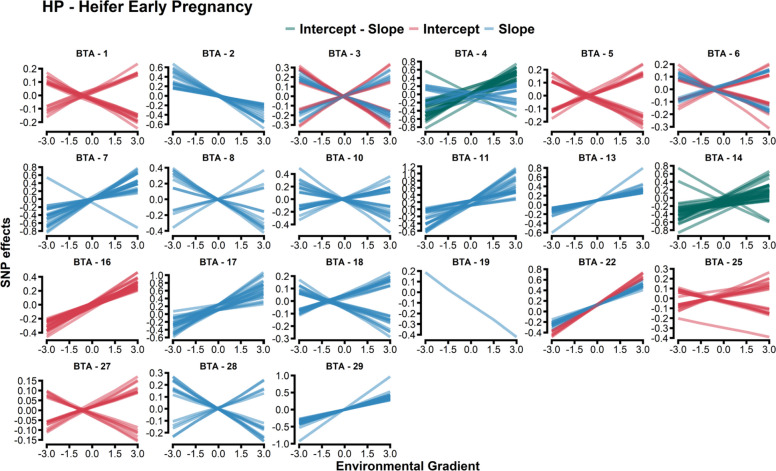
Predicted SNP effects across the environmental gradient (EG) for heifer early pregnancy (HP), based on significant SNPs $\left(-\!\log_{10}(P\mathrm{-value})>5\right)$ in the GWAS of reaction norm parameters for HP. Colors indicate SNPs associated with the intercept (blue), slope (red), or both reaction norm parameters (green). These trajectories are descriptive visualizations derived from the reaction norm coefficients

**Fig. 6:**
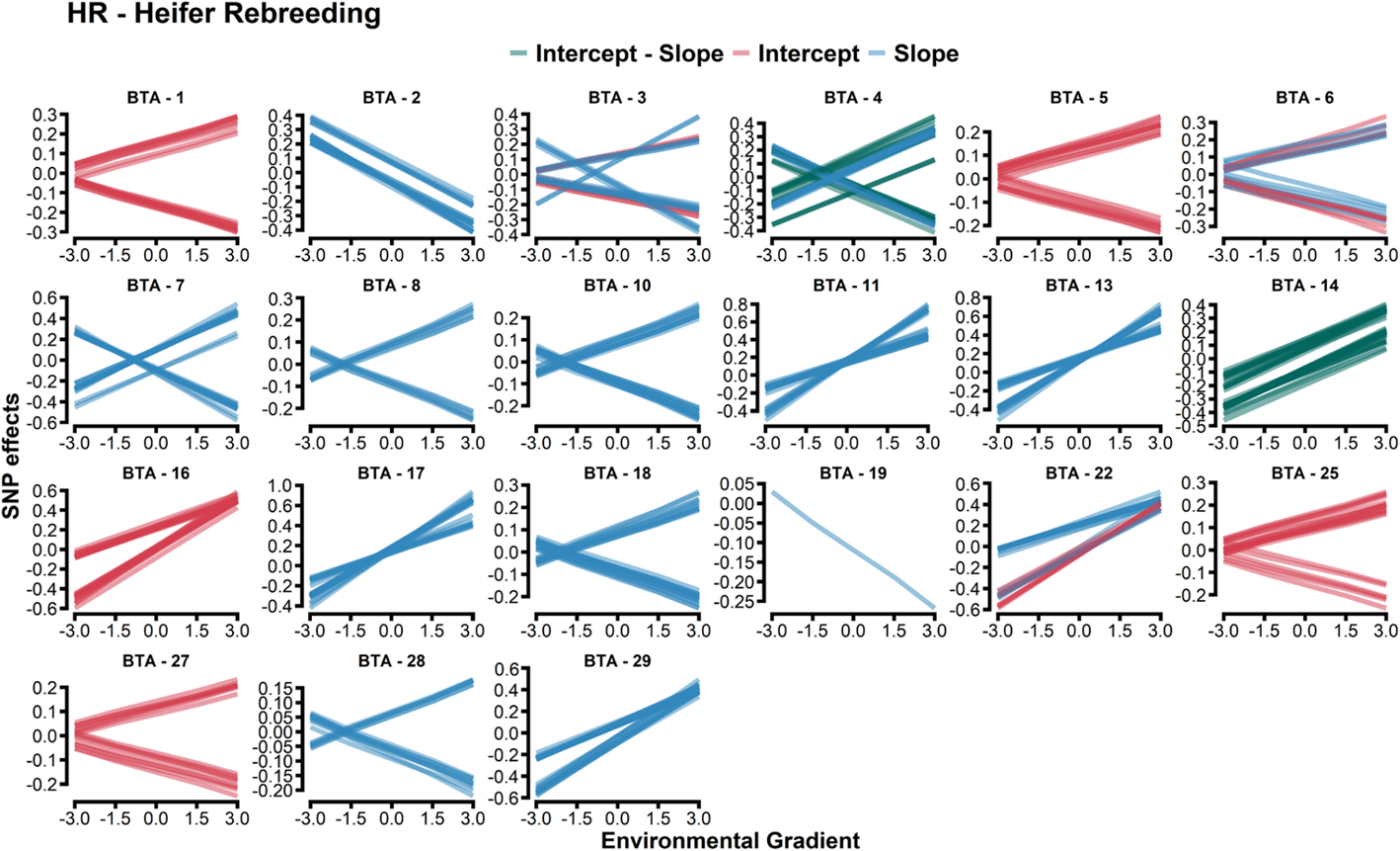
Predicted SNP effects across the environmental gradient (EG) for heifer rebreeding (HR), based on SNPs deemed as significant $\left(-\!\log_{10}(P\mathrm{-value})>5\right)$ in the GWAS of reaction norm parameters for HR. Colors indicate SNPs associated with the intercept (blue), slope (red), or both reaction norm parameters (green). These trajectories are descriptive visualizations derived from the reaction norm coefficients

Across the EG levels, predicted SNP effects for significant loci in the GWAS of RN parameters varied in magnitude and in some cases, direction, consistent with SNP environment-dependent effects (Figs. 5 and 6). HP showed more heterogeneous SNP-effect trajectories than HR, whereas HR exhibited comparatively more stable patterns across the environmental gradient. In general, predicted additive SNP effects tended to be greater under more favorable EG conditions, in agreement with the increase in additive genetic variance observed for both traits (Fig. 3).

#### Genomic regions controlling the reproductive potential (intercept)

Significant genomic regions associated with the intercept were mapped on BTAs 1, 3, 5, 6, 16, 22, 25, and 27 (Fig. 4a). These loci explained a considerable proportion of the total additive genetic variance (Tables 1 and 2; see Additional file 2: Figs. S4a and S5a). Furthermore, moderate LD (*r*^2^) was observed, with average values ranging from 0.30 (BTA1) to 0.47 (BTA27; see Additional file 2: Fig. S6), indicating stable genetic signals across EG levels. Fine-mapping using imputed whole-genome sequence data prioritized variants with high posterior inclusion probabilities (PIP > 0.90; ${\mathrm{log}}_{10}\left(BF\right)=3.5$). These variants clustered within loci harboring genes related to neuroendocrine regulation and reproductive axis activation, including *IGF1*, *GHRL, LEP, PMHC, GNRHR, PLAG1, KISS1, MAPK3* and *GHRL* (see Additional file 2: Fig. S7).

On BTA1, a gene set (*ASTE1, ATP2C1, BRWD1, GET1, SH3BGR, LCA5L, B3GALT5, IGSF5, PCP4,* and *DSCAM)* collectively explained 1.76% (HP) and 2.12% (HR) of the total additive genetic variance and were associated with neuronal differentiation and calcium signaling pathways (Tables 1 and 2). The BTA3 region (29.14–29.88 Mb*, OLFML3, HIPK1* and *PTPN22*) explained 0.95% for HP and 1.35% for HR of the total additive genetic variance (Tables 1 and 2) and was linked to immune and stress-related regulatory functions. The genomic region on BTA5 (66.43–66.52 Mb) harbored *IGF1, PMCH,* and *PARPBP*, and *U6*, which modulate follicular growth and luteal competence affecting reproductive performance (Tables 1 and 2) accounted for 0.95% to 2.16% of the total additive genetic variance of HP and 0.95% to 2.29% for HR. The BTA6 (83.15–85.17 Mb) harbored *GNRHR*, *STAP1*, and *SULT1B1*, reinforcing roles in GnRH signaling and hormone metabolism, explaining up to 0.96%–2.98% (HP) and 0.99%–1.82% (HR) of the total additive genetic variance of the respective traits.

BTA16 (0.81–2.01 Mb) included three regions harboring *KISS1, ZBED6, ADORA1, PLEKHA6*, and other regulators of puberty onset, muscle development, and metabolic control, accounting for considerable genetic variance of 0.99%–3.29% for HP and 0.97%–2.57% for HR. BTA22 (41.12–55.11 Mb) spanned *GHRL, HRH1, ATP2B2,* and *FHIT*, with variance explained from 0.95% to 3.52% for HR and 0.96% to 3.05% for HP. The genes located in this region are associated with feeding behavior, calcium signaling, and energy homeostasis. BTA25 (25.25–26.92 Mb) emerged as a highly gene-dense region with 65 significant SNPs encompassing *MAPK3, SH2B1, CD19, ATP2A1* and multiple zinc-finger and immune-related genes. This region accounted for 0.99%–1.98% of HP and 0.97%–1.78% of HR’s additive genetic variance, highlighting MAPK cascade activation, hormone secretion, and immune signaling under environmental modulation. Finally, BTA27 (16.22–16.61 Mb) included *TLR3, MTNR1A,* and *F11*, which are genes linked to innate immunity and reproductive seasonality, explaining 0.99%–2.36% and 0.95%–2.39% of the additive genetic variance for HP and HR, respectively. These results underscore the polygenic and environment-sensitive architecture underlying early reproduction in Nellore heifers, pinpointing central nodes of the hypothalamic–pituitary–gonadal (HPG) axis, stress-induced immune response, and metabolic flexibility in the adaptation to tropical environments.

#### Genomic regions underlying environmental sensitivity (slope)

A multi-trait GWAS summary identified 36 genomic regions with significant SNP × E interactions distributed across 16 autosomes (BTAs 2, 3, 4, 6, 7, 8, 10, 11, 13, 14, 17, 18, 19, 22, 28, and 29; Fig. 4b). These regions captured genotype-specific plasticity, with the greatest differentiation in SNP effects observed under favorable EG levels (Figs. 5 and 6) and explained the largest proportion of the total additive genetic variance (Table 3; see Additional file 2: Figs. S4b and S5b). LD within these genomic regions was moderate to high (*r*^2^ = 0.31–0.60), indicating the presence of co-inherited haplotype blocks modulating environmental sensitivity (see Additional file 2: Fig. S8). Allelic substitution effects varied markedly across EG levels, indicating SNP × E-dependent changes in animal environmental responsiveness (Figs. 5 and 6). Fine-mapping analysis highlighted variants in key endocrine and metabolic regulators, including *INS–IGF2* (BTA29)*, INSR* (BTA7)*, IGFBP2/IGFBP5* (BTA2)*, FSHR, LHCGR, ESR2* and *EGFR* (see Additional file 2: Fig. S9).

The proportion of the total additive genetic variance explained by individual loci ranged from 0.42% (BTA19) to 4.07% (BTA29) for HP and from 0.46% (BTA19) to 5.34% (BTA14) for HR (see Additional file 2: Figs. S4 and S5; see Additional file 1: Table S2). Several regions mapped to biologically relevant genes involved in endocrine signaling, metabolic control, and neuroendocrine regulation. On BTA2, a locus harboring *IGFBP2* and *IGFBP5* explained 1.09%–2.29% of the variance in HP and 1.01%–2.75% in HR. BTA3 contained multiple consecutive regions, including one enriched for *HSD17B7*, *RGS4*, and *RGS5*, explaining up to 2.91% of the additive genetic variance of HR.

On BTA6, adjacent loci spanning 64.93–65.91 Mb included *GABRA2*, *GABRA4*, and *GABRB1*, genes related to GABAergic signaling and potential modulation of the HPG axis. Multiple genomic regions on BTA7 are associated with energy balance and immune signaling. The region harboring *INSR* (16.09–16.19 Mb) explained 3.15%–3.62% for HP and 2.28%–3.47% for HR, while regions mapping *ANGPTL4*, *TNFSF9*, *CD70*, and *TRIP10*, associated with both metabolic and immune modulation, explained from 0.98% to 3.64% of the total additive genetic variance. On BTA10, (76.38–76.47) *ESR2* and *MTHFD1* genes exhibited clear SNP×E dependent effect switches, with *ESR2* shifting from negative effects at EG = −3.0 to positive at EG = 3.0, explaining 1.28%–2.6% (HP) and 2.1%–3.0% (HR).

SNPs on BTA11 mapped reproductive receptor genes *LHCGR* and *FSHR* explained 1.07%–2.65% of the genetic variance in HP and 1.01%–2.64% in HR. BTA13 encompassed five adjacent genomic regions, surrounding the most prominent gene set (*GHRH*, *SRC*, and *SAMHD1*) explaining between 1.69%–2.87% of the total additive genetic variance and showed strong SNP×E interactions (Figs. 5 and 6), in addition to contributing to reproductive plasticity (Fig. 3). The region on BTA17 (41.11–41.98 Mb), which includes the *GRIA2* and *GLRB* genes, related to regulation of hormonal cycles and reproductive behaviors showed consistent environmental responsiveness (Figs. 5 and 6), accounting for up to 2.49% of the additive genetic variance in HR. On BTA22, the region spanning 0.81–0.96 Mb, which contains *EGFR* and *LANCL2*, explained 1.00%–1.80% (HP) and 2.88%–3.81% (HR). The SNPs within the *RYR2* gene on BTA28 (10.10–10.39 Mb) showed a distinct shift in allelic effects around EG = 0 for HP and EG = 1.5 for HR, contributing up to 3.50% (HP) and 3.54% (HR).

The strongest G × E interaction signal was detected in a region on BTA29 (48.90–49.49 Mb), encompassing the imprinted genes *INS* and *IGF2*. These SNPs showed marked shifts in allelic effects across the EG, from negative under harsh conditions to positive in favorable environments and explained up to 4.07% (HP) and 1.73% (HR) of the total additive genetic variance of the respective traits. The high LD within this region (mean *r*^2^ = 0.52) supports the presence of cis-regulatory elements controlling environmentally responsive expression, emphasizing the central role of the insulin–IGF signaling axis in linking metabolic state to reproductive performance.

#### Shared genomic regions link genetic potential and plasticity

Shared SNPs on BTA4 and BTA14 (Fig. 4) influenced both intercept and slope, indicating pleiotropic control of genetic potential (intercept) and environmental responsiveness (slope) of reproductive performance (HP and HR; Tables 1 and 3). LD analysis indicated a strong signal around the top-scoring SNP, with average *r*^2^ values of 0.37 (intercept) and 0.34 (slope) on BTA4 and 0.41 on BTA14 for both parameters (see Additional file 2: Figs. S6 and S8). SNP effect sizes increased progressively across the EG (Figs. 5 and 6), highlighting the environment-dependent contribution of these loci to reproductive performance, particularly under more favorable environmental conditions. Markers within BTA4, explaining 0.95%–3.87% of the additive variance, included genes related to metabolism and immune regulation (*LEP, HILPDA, SND1, RBM28* and *IRF5*). On BTA14, variants explained up to 5.34% of the total additive genetic variance and harbored key growth and fertility regulators (*PLAG1, MOS, LYN, PENK, RPS20, CHCHD7*, and *SDR16C5*). These findings highlight a set of pleiotropic loci that influence both baseline reproductive potential and plasticity across EGs, providing a functional basis for G × E in Nellore cattle.

### Fine mapping prioritizes candidate variants and biologically plausible genes

Fine-mapping refined GWAS signals to smaller sets of statistically prioritized variants, indicating that within each associated region a limited subset of variants captured most of the posterior support under the model (see Additional file 2: Figs. S6 and S8). These variants mapped to genes involved in endocrine and metabolic regulation relevant to heifer fertility in tropical production systems.

For the RN intercept (baseline reproductive genetic merit), fine-mapping highlighted candidate variants within loci mapping the *IGF1* and *PMCH* (BTA5), *GNRHR* (BTA6), *KISS1* (BTA16), and *GHRL*/energy homeostasis-related genes (BTA22), consistent with a genetic architecture linking growth, energy balance, and neuroendocrine regulation of reproduction (see Additional file 2: Fig. S7). For the RN slope (environmental responsiveness), the top-prioritized variants were observed in loci related to metabolic regulation and endocrine signaling, including the insulin–IGF axis (*INS*–*IGF2* at BTA29; *INSR* at BTA7; *IGFBP2*/*IGFBP5* at BTA2), and gonadotropic signaling (*FSHR* and *LHCGR*), suggesting that variation in reproductive plasticity may involve differences in sensitivity to metabolic status and hormonal feedback (see Additional file 2: Fig. S9). Overall, fine-mapping provided a statistical refinement of the meta-GWAS loci and enabled prioritization of candidate variants and nearby biologically plausible genes for future functional validation of baseline fertility potential and resilience-related responsiveness across environmental gradients.

### Functional enrichment links fertility potential and environmental resilience

Gene enrichment analyses characterize distinct but interconnected biological networks. Intercept-associated genes were enriched in neuroendocrine regulation, immune signaling and energy metabolism, whereas slope-associated genes emphasized endocrine feedback, immune plasticity and metabolic adaptability (Tables 4, 5 and 6). Together, these results support a model in which reproductive performance emerges from the integration of baseline genetic merit and environmentally modulated physiological plasticity.

**Table 4:** Gene Ontology (GO) enrichment analysis for biological processes of the genes identified for the intercept of reaction norm model using the multi–trait meta–analysis statistical test for heifer early pregnancy (HP) and heifer rebreeding (HR)

GO ID	Description	*P*-value	Gene ID
Immune System Regulation			
GO:0050855	Regulation of B cell receptor signaling pathway	0.0001	*PTPN22, STAP1, LYN, CD19*
GO:0050863	Regulation of T cell activation	0.0026	*PTPN22, LEP, IGF1, LYN, LAX1, SOX13, LAT, IL27, CORO1A, SPN*
Reproductive and Hormonal Regulation			
GO:0060986	Endocrine hormone secretion	0.0002	*LEP, GNRHR, REN, KISS1, GHRL*
GO:0032274	Gonadotropin secretion	0.0005	*LEP, GNRHR, KISS1*
GO:0032275	Luteinizing hormone secretion	0.0042	*LEP, KISS1*
GO:0042445	Hormone metabolic process	0.0222	*BACE2, LEP, SULT1B1, SDR16C5, REN, SULT1A1*
Neural and Synaptic Regulation			
GO:0032228	Regulation of synaptic transmission, GABAergic	0.0003	*ADORA1, ZDHHC3, SLC6A1, CLN3*
GO:0007631	Feeding behavior	0.0163	*LEP, PMCH, REN, GHRL*
GO:0032355	Response to estradiol	0.0206	*LEP, PENK, MYOG, SLC6A1*
Metabolic and Cellular Processes			
GO:0043410	Positive regulation of MAPK cascade	0.0013	*PTPN22, LEP, KLHDC10, IGF1, MOS, ADORA1, CHI3L1, KISS1, GHRL, MAPK3, TAOK2, TLR3*
GO:0051403	Stress–activated MAPK cascade	0.0105	*KLHDC10, MAPK3, TAOK2*
GO:0032107	Regulation of response to nutrient levels	0.0264	*LEP, GHRL*
Growth and Muscle Development			
GO:0060123	Regulation of growth hormone secretion	0.0051	*KISS1, GHRL*
GO:0003012	Muscle system process	0.0091	*LEP, IGF1, MYOG, ADORA1, MYBPH, ATP2B4, GHRL, ATP2A1, ALDOA, MYL11*

**Table 5:** Gene Ontology (GO) enrichment analysis for biological processes of the genes identified for the slope of reaction norm model using the multi-trait meta-analysis statistical test for heifer early pregnancy (HP) and heifer rebreeding (HR)

GO ID	Description	*P*-value	Gene ID
Immune System Regulation			
GO:0050870	Positive regulation of T cell activation	5.4 × 10^–5^	*TNFSF9, CD70, VAV1, LYN, LEP, IGF2, IGFBP2, BRD7, SRC, TNFSF14*
GO:0002696	Positive regulation of leukocyte activation	5.5 × 10^–5^	*TNFSF9, CD70, VAV1, LYN, LEP, IGF2, IGFBP2, BRD7, CD320, LBP, SRC, TNFSF14*
Reproductive and Hormonal Regulation			
GO:0042698	Ovulation cycle	3.1 × 10^–5^	*LEP, GABRB1, EGFR, FSHR, LHCGR, SRC*
GO:0001541	Ovarian follicle development	0.01208	*FSHR, LHCGR, SRC*
GO:0043434	Response to peptide hormone	8.2 × 10^−5^	*LYN, KCNQ1, TH, INS, LEP, MTCL2, IGF2, INSR, CTSD, IGFBP5, LHCGR, DDR2, SRC*
Neural and Synaptic Regulation			
GO:0050808	Synapse organization	0.00092	*GPHN, INS, NOS1AP, INSR, GABRA4, ASAP1, GABRA2, DBNL, LRRC4, GLRB, ACTL9, C3*
GO:0007214	Gamma–aminobutyric acid signaling pathway	0.00178	*GABRA4, GABRB1, GABRA2*
Metabolic and Cellular Processes			
GO:0006006	Glucose metabolic process	0.00021	*KCNQ1, INS, LEP, MTCL2, IGF2, INSR, PGAM2, SRC*
GO:0032868	Response to insulin	0.00044	*LYN, KCNQ1, INS, LEP, MTCL2, IGF2, INSR, CTSD, SRC*
GO:0043410	Positive regulation of MAPK cascade	0.00026	*MOS, INS, LEP, IGF2, INSR, EGFR, FSHR, NKD1, KLHDC10, SRC*
GO:0010883	Regulation of lipid storage	0.00107	*HILPDA, LEP, C3, ALKBH7*
GO:0006112	Energy reserve metabolic process	0.00663	*INS, LEP, IGF2, INSR*
Growth and Muscle Development			
GO:0042692	Muscle cell differentiation	0.02776	*FLNC, MYL9, IGF2, SMO, IGFBP5, RGS4, TNNT3, TNFSF14*
GO:0045927	Positive regulation of growth	0.0060	*INS, LEP, IGF2, SMO, INSR, EGFR, GHRH*
GO:0034103	Regulation of tissue remodeling	0.00612	*LEP, EGFR, DDR2, SRC*

#### Gene enrichment for intercept

Genes determining genetic potential (intercept) for HP and HR were enriched in immune regulation, endocrine function, and energy metabolism functions (Table 4). Notably, *PTPN22* was involved in adaptive immunity through B and T cell signaling (GO:0050855, GO:0050863). Endocrine secretion pathways were overrepresented by the genes *LEP*, *GNRHR*, and *KISS1*, which affect endocrine hormone secretion (GO:0060986), gonadotropin secretion (GO:0032274), and luteinizing hormone secretion (GO:0032274). Complementing these biological GO, neuroendocrine regulation through GABAergic synaptic transmission (GO:0032228) and feeding behavior (GO:0007631), by the action of the major gene set (*PMCH, ADORA1* and *SLC6A1*) suggesting neuroendocrine control of puberty and energy balance. Genes such as *IGF1*, *MAPK3*, and *TAOK2* connected MAPK signaling (GO:0043410 and GO:0051403) and nutrient sensing (GO:0032107) to reproductive control. These results support a neuroendocrine–metabolic network modulating genetic potential in reproduction (Table 4).

Pathway analysis supports the hypothesis that high genetic merit for fertility is closely linked to a complex metabolic-neuroendocrine network (Table 6). Major signaling pathways cascade included leptin signaling (R-BTA-2586552), ghrelin processing (R-BTA-422085), and GnRH pathway (bta04912) converging on progesterone-mediated oocyte maturation (bta04914). Additionally, energy-sensing and proteostatic mechanisms such as glucose metabolism (R-BTA-70326), MAPK (bta04010), JAK-STAT (bta04630), and heat shock response (R-BTA-3371453) were also found to play a complementary role in regulating genetic potential of reproductive performance (intercept) (Table 6).

**Table 6:** Gene enrichment analysis for Reactome and KEGG pathways, related to gene set identified for the intercept and slope of reaction norm model using the multi-trait meta-analysis statistical test for heifer early pregnancy (HP) and heifer rebreeding (HR)

Path ID	Description	*P*-value	Gene ID
Intercept			
Reproduction and Sexual Precocity			
R–BTA–2586552	Signaling by Leptin	0.0050	*LEP, SH2B1*
R–BTA–422085	Synthesis, secretion, and deacylation of Ghrelin	0.0008	*LEP, IGF1, GHRL*
R–BTA–416476	G alpha (q) signaling events	0.0196	*PMCH, GNRHR, KISS1, GHRL, HRH1, MAPK3*
bta04912	GnRH signaling pathway	0.0265	*GNRHR, MAPK3*
bta04914	ProgesteronE-mediated oocyte maturation	0.0315	*IGF1, MOS, MAPK3, KIF22*
Indirectly Related to Sexual Precocity and Reproduction			
R–BTA–70326	Glucose metabolism	0.0396	*NUP37, SEC13, ALDOA*
R–BTA–3371453	Regulation of HSF1–mediated heat shock response	0.0303	*NUP37, SEC13, MAPK3*
bta04935	Growth hormone synthesis, secretion and action	0.0441	*IGF1, GHRL, MAPK3*
bta04630	JAK–STAT signaling pathway	0.0371	*LEP, IL27, CTF1*
bta04010	MAPK signaling pathway	0.0463	*IGF1, MAPK3, TAOK2*
Slope			
Reproduction and Sexual Precocity			
bta04913	Ovarian steroidogenesis	1.6 × 10^–5^	*INS, INSR, ADCY7, FSHR, HSD17B7, LHCGR*
bta04915	Estrogen signaling pathway	0.015	*ADCY7, CTSD, EGFR, ESR2, SRC*
bta04917	Prolactin signaling pathway	0.001	*TH, INS, ESR2, LHCGR, SRC*
Modulate Growth, Metabolism and Development			
R–BTA–74752	Signaling by Insulin receptor	0.001	*INS, INSR, CTSD, ATP6V1F, ATP6V1D*
R–BTA–9843745	Adipogenesis	0.019	*LEP, ZNF423, TGS1, ANGPTL4*
bta04010	MAPK signaling pathway	0.041	*INS, IGF2, INSR, DUSP8, EGFR*
bta04014	Ras signaling pathway	0.048	*INS, IGF2, INSR, EGFR*
bta04152	AMPK signaling pathway	0.007	*INS, LEP, INSR, RAB11B*
bta04727	GABAergic synapse	6.6 × 10^–5^	*GPHN, ADCY7, GABRA4, GABRB1, GABRA2, SRC*
bta04081	Hormone signaling	3.4 × 10^–6^	*INS, LEP, IGF2, INSR, ADCY7, FSHR, PENK, ESR2, GHRH, LHCGR, SRC*

#### Gene enrichment for environmental responsiveness (slope)

Genes associated with the slope (environmental responsiveness) of ssGRN for HP and HR, exhibited enrichment for immune activation, endocrine feedback, and metabolic adaptation (Table 5). GO terms such as positive regulation of T cell activation (GO:0050870) and leukocyte activation (GO:0002696), including the major genes *TNFSF9*, *VAV1*, *LYN*, *LEP*, *IGF2* and *SRC*, suggest enhanced immune plasticity under variable conditions. Reproductive processes such as ovulation cycle (GO:0042698), ovarian follicle development (GO:0001541), and response to peptide hormone (GO:0043434), involved the major genes *LEP*, *GABRB1*, *EGFR*, *FSHR*, *LHCGR*, *INS*, *IGF2*, *INSR* and *IGFBP5*. These results indicate that slope-associated genes modulate ovarian activity in response to environmental signals.

Metabolic adaptability plays a crucial role in regulating reproductive efficiency in response to EG changes (Table 5). Supported by glucose metabolic process (GO:0006006), response to insulin (GO:0032868), regulation of lipid storage (GO:0010883), MAPK regulation (GO:0043410), and energy reserve metabolic process (GO:0006112) driven by *INS, LEP, IGF2, INSR*, and *HILPDA*. Synaptic pathways (GO:0050808, GO:0007214) included *GABRA2*, *GABRB1*, *GPHN*, and *GLRB*, suggesting neurotransmitter-mediated modulation of reproductive responses to environmental stress.

Pathway enrichment analyses provided a clearer understanding of the functional roles of slope-associated genes (Table 6). Core reproductive pathways included ovarian steroidogenesis (bta04913), estrogen signaling (bta04915), and prolactin signaling (bta04917), driven by *INS*, *FSHR*, *ESR2*, *EGFR*, and *LHCGR*. Energy-sensing pathways such as insulin signaling (R-BTA-74752), AMPK signaling (bta04152), adipogenesis (R-BTA-9843745), and the Ras/MAPK cascade (bta04010, bta04014) are linked with energy availability to reproductive plasticity. GABAergic synapse pathways (bta04727) reinforced the connection between environmental perception and neuroendocrine processes.

## Discussion

Understanding how genetic merit changes across environmental contexts is essential for accurately estimating genetic parameters and designing robust breeding strategies, particularly for fertility traits whose expression is tightly constrained by energy balance and heat load [1, 5]. By modeling both RN intercept (baseline genetic merit) and slope (environmental sensitivity) for HP and HR, we observed a distinct biological mechanism through which G × E modulates reproductive performance in Nellore cattle.

The intercept and slope parameters capture complementary biological mechanisms controlling reproductive efficiency (HP and HR). The intercept represents the expected genetic merit at the reference environment and, therefore, concentrates loci and pathways that regulate neuroendocrine activation and energy-gated reproductive competence. The slope quantifies genotype-specific responsiveness to environmental improvement and captures plasticity mechanisms that translate additional resources into reproductive activation and recovery. This separation has direct implications for selection in tropical systems, genotypes that rank highly under favorable conditions can be disproportionately environment-dependent, whereas genotypes with favorable slopes can maintain, or improve, performance as conditions improve, thereby supporting both productivity and resilience.

### Heritability estimates

Reproductive efficiency (HP and HR) is complex and regulated by multiple physiological pathways, making them particularly sensitive to environmental stressors [9, 52]. G × E interactions add a further layer of complexity by inducing environment-specific genetic variance, thereby the omission of this effect in the genetic evaluation models reduces the accuracy of (G)EBV predictions and selection responses [53, 54]. Our results confirm that genetic variance for HP and HR is environment-specific (Fig. 1a and b). Heritability estimates increased progressively from harsh to favorable EG, following a sigmoidal trend consistent with previous reports [5, 11, 17]. In harsh environments, genetic variance is suppressed, reducing phenotypic expression and heritability, whereas under intermediate (−2.5 < EG < 2.5) and favorable conditions, additive genetic variance increases, improving genetic expression and the proportion of phenotypic variance explained by genetics as well as improving selection efficiency [55].

This dynamic pattern supports strong G × E effects on reproductive traits (Fig. 1a and b). At medium EG level, the estimated heritability for HP (*h*^2^ = 0.30 ± 0.02) aligns with previous reports in Nellore heifers without G × E modeling (*h*^2^ = 0.28–0.31) [9, 56]. The heritability for HR (*h*^2^ = 0.33 ± 0.03) exceeds BLUP-based estimates (0.21–0.31) for the same population [1, 57], suggesting that the inclusion of G × E captures part of the genetic variance that would otherwise be attributed to residual effects. These results emphasize the importance of modeling G × E interactions in genetic evaluations. In resource-rich environments, reproductive performance can be effectively expressed, whereas in harsher conditions, marked by nutritional inadequacies or heat stress, environmental noise can hinder genetic potential. This makes the identification of resilient genotypes essential for stable reproductive performance [58].

### Genetic correlation across environments and reaction norm parameters

G × E interactions markedly affect HP and HR, especially in extensive tropical production systems. Genetic correlations between EG levels demonstrated an environment-specific expression of genetic merit, with a more pronounced G × E effect under challenging conditions characterized by heat stress and nutritional restriction, which can impair reproductive performance [27, 29]. Notably, HR exhibited greater variability in genetic correlations across EG than HP, indicating higher environmental sensitivity. This reflects the intensified physiological demands placed on primiparous females, who need to manage growth, lactation, and reproductive recovery under stressful conditions.

Genetic correlations remained above 0.80 when EG levels were similar (Fig. 1c and d), suggesting a lower probability of genetic merit reranking [11]. However, as differences in EG levels increase, genetic correlations, decreased below 0.80, especially for HR, highlighting a notable G × E effect with reranking [27, 59]. This suggests that animals selected under favorable conditions may not sustain equivalent reproductive performance under harsher EGs [60]. Similar patterns have been reported for HP by Mota et al. [5] (0.30 to 0.99) and Santana et al. [61] (0.10 to 1.0). High reproductive performance under favorable conditions may not translate in greater resilience under harsher environmental conditions.

Genetic correlations between the intercept and slope parameters in the RN models illustrate the architecture of reproductive plasticity. For HP (*r*_*g*_ = 0.71; Fig. 2a) and HR (*r*_*g*_ = 0.50; Fig. 2b) these correlations indicate partial genetic overlap between reproductive potential and environmental responsiveness. These genetic correlations reflect that selection for increased reproductive performance under average conditions (intercept) may also enhance responsiveness to improved environments (slope). This implies that selecting animals with high genetic merit (intercept) and adaptive plasticity (slope) can enhance reproductive efficiency across variable environmental conditions [54, 62, 63] and supports previous evidence of genotype reranking under heat stress and nutritional challenges [5, 17, 27, 64].

Pearson correlation of GEBVs between HP and HR at the intercept was moderate (*r* = 0.61; Fig. 2c), suggesting a partial genetic overlap, where animals genetically superior for HP tend to perform well for HR. However, trait-specific components remain, as HP is more related to pubertal onset, whereas HR reflects postpartum recovery and ovarian cyclicity. Conversely, the stronger correlation observed for the slope parameter (*r* = 0.73; Fig. 2d) indicates a shared genetic basis for environmental responsiveness. Specifically, animals that exhibit greater increases in HP as the environment becomes more favorable also increases the probability of HR across the same environmental gradient, suggesting a shared regulatory architecture for adaptive reproductive responses, such as energy metabolism, neuroendocrine signaling, and immune regulation [9].

### Reaction norm and sire reranking across environments

Our results show that G × E interaction influences HP and HR in Nellore cattle under tropical beef production conditions (Fig. 3). The EG, derived from the BLUE solution for YW, encompasses between-herd-year variation in post-weaning nutritional supply and management intensity, thereby reflecting the environmental conditions that shape female growth and physiological development [14, 65]. Within this framework, greater HP would be expected in more favorable environments that support adequate post-weaning growth and allow heifers to reach an appropriate body weight (55%–65% of mature weight) at the beginning of the breeding season [1, 66, 67]. Additionally, higher HR is likely favored when these same environments support not only appropriate development before first mating, but also the maintenance of body condition and energy reserves after calving, which are essential for postpartum recovery, resumption of ovarian cyclicity, and successful rebreeding [68]. Therefore, the more positive EG values observed in this study (0 to 3) probably represent production contexts in which heifers are better able to express their genetic potential for earlier puberty and successful rebreeding. This interpretation is consistent with evidence showing that nutritional management and growth rate are major drivers of puberty attainment in Nellore heifers and that body weight and body condition around calving are closely associated with postpartum reproductive success [67].

Sire reaction norms (Fig. 3a and c), together with genetic correlations below 0.80 between contrasting EG levels, show clear changes in GEBV trajectories across environments, leading to reranking between less favorable and more favorable conditions. Thus, sires identified as superior under high EG do not necessarily maintain the same advantage under low EG. This is the main practical implication of model choice: a conventional BLUP assumes the same breeding value across environments, whereas the RN model captures both average genetic merit and environmental sensitivity. In beef cattle, the key issue is whether animal ranking changes across production environments, and ignoring this may decrease selection efficiency when heifers are raised in conditions different from those in which animals fully express their genetic potential [5]. From a practical breeding perspective, individuals should be selected according to the target production environment, preferably using environment-specific GEBVs from RN models rather than a single BLUP evaluation. For low-input systems, selecting individuals under low EG (EG < 0) is more appropriate, whereas selection under medium EG may be an effective strategy for identifying individuals with robust performance in improved environments (EG from 0 to 3), given the greater ranking stability observed between medium and high EG levels (Fig. 3b and d). In breeding programs targeting heterogeneous systems, priority should be given to robust individuals that combine high genetic merit for the target traits with lower environmental sensitivity [11, 12, 69].

The practical relevance of this reranking is reinforced by the limited overlap among top-ranking sires across EG levels (Fig. 3b and d). Of the 90 sires ranked for HP and HR, only 13 and 10 sires, respectively, were common across low, medium, and high EG levels. These results highlights the challenge of identifying broadly superior sires for use in heterogeneous production systems [70]. Rankings were more stable between medium and high EG levels, suggesting greater similarity between these environments than between either of them and low EG (Fig. 3b and d). Indicating that selection under medium EG conditions may also be effective for identifying sires with robust performance in improved environments, whereas performance under low EG was less predictive of ranking in more favorable conditions.

The positive correlation between intercept and slope for HP and HR (Fig. 2a and b) indicates that animals with higher genetic merit also tend to benefit more from improved environments (EG > 0). The increase in mean GEBVs from low to high EG levels for both HP (from 57.99 ± 6.79 to 77.96 ± 9.44, Fig. 3e) and HR (from 55.65 ± 2.87 to 71.98 ± 4.1, Fig. 3f) reinforces the role of EG in modulating genetic expression. While GEBVs are theoretically centered at zero on the liability scale, this assumption does not hold after transformation to the probability scale, particularly when results are stratified by environmental gradient. The observed shift reflects not a change in genetic merit, but rather the differential expression of that merit under varying environmental conditions. Additionally, the variability in individual GEBV trajectories across EG levels reveals differences in environmental plasticity among sires. These results reinforce the importance of considering both genetic merit and environmental responsiveness in selection. Traditional BLUP models that ignore G × E may lead to suboptimal animal selection when the evaluation environment differs from commercial production environments, underestimating the expected genetic gain. Integrating RN models into selection strategies and prioritizing animals with both high performance and favorable responsiveness will enhance reproductive efficiency and herd resilience under tropical conditions, supporting sustained genetic progress amid climate variability and resource constraints.

### Mapping of genomic regions related to reaction norm parameters

GWAS and fine-mapping analyses identified genomic regions associated with the reaction norm (RN) parameters of HP and HR in Nellore cattle. Because linkage disequilibrium can hinder the direct identification of causal variantseta-GWAS signals, locus-specific LD patterns, and imputed whole-genome sequence (WGS) data reduced the number of plausible candidate variants and highlighted the most relevant genomic regions. These regions mapped genes with putative effects on the intercept, mainly related to neuroendocrine activation and energy-dependent reproductive competence (*IGF1, PMCH*, *KISS1*, *GNRHR*, *GHRL* and *LEP*). On the other hand, RN slope loci emphasized genes involved in metabolic and endocrine plasticity (*INS*–*IGF2*, *INSR*, *IGFBP2, IGFBP5*, *FSHR* and *LHCGR*), consistent with genotype-specific responsiveness to environmental improvement [16, 71].

These findings provide a framework for environment-informed selection by distinguishing genomic regions associated with genetic potential for reproductive performance (intercept) from those associated with environmental sensitivity (slope). Fine mapping of these regions identified credible sets that reduce the number of candidate variants underlying each association signal. This may help prioritize loci functional annotation, replication in independent populations, and subsequent gene expression. However, statistical fine mapping does not establish biological causality, and validation in independent populations, functional genomic validations, and relevant functional contexts remains necessary before stronger biological or breeding inferences can be made.

#### SNP effects across environmental gradient

Our results show a complex genetic architecture that supports reproductive performance (HP and HR) in Nellore cattle. The correlation between HP and HR was moderate for the intercept (*r* = 0.61; Fig. 2c) and moderate to high for the slope (*r* = 0.73; Fig. 2d). Using a biologically informed approach to combine GWAS results [32], we identified 482 significant SNPs for the intercept and 700 for the slope of HP and HR. Among these, 131 SNPs on BTA4 (92.38–93.81 Mb) and BTA14 (23.03–24.06 Mb) exhibited pleiotropic effects, suggesting partial overlap between the animal’s genetic potential (intercept) and environmental sensitivity (slope). These regions harbor genes involved in the hypothalamic–pituitary–ovarian axis (Tables 1, 2 and 3), a key neuroendocrine pathway regulating puberty onset and postpartum ovarian reactivation [72] and important for HP and HR.

Significant SNP markers exhibited clear SNP×E interaction, with marker effects varying across EGs (Figs. 5 and 6). In some cases, both the magnitude and direction of allelic effects changed across environments (Fig. 5), indicating that the contribution of these loci is environment-dependent [8]. Under more challenging conditions, SNP × E may alter the relative contribution of loci to phenotypic expression [8, 73], potentially leading to reranking of genetic merit. Similar patterns have been reported in swine [74], dairy cattle [17], and beef cattle [5, 11, 73].

The SNP×E interactions identified in our study were characterized by variation in both the magnitude and direction of allelic effects across environments (Fig. 5), indicating environment-dependent SNP effects [8, 64, 73]. This environment-dependent allelic response is particularly relevant when variants are located near genes regulating key physiological processes, as they modulate both phenotypic expression and the distribution of genetic variance across environments [5, 8, 73]. Additive SNP effects for HP and HR were generally greater in favorable environments (Fig. 5a and b), whereas some chromosomes showed less variation in SNP effects at intermediate EG levels. On the other hand, some chromosomes exhibited less variability in SNP effects at medium EG levels. These results reinforce the importance of considering environmental changes in genomic evaluations. Ignoring this variation may bias selection toward genotypes with advantages restricted to particular conditions [75]. Accounting for SNP×E may therefore improve the identification of genotypes with broader environmental stability or more specific adaptive responses.

#### Genomic regions with effect on intercept

Genomic regions associated with the intercept for HP and HR indicate that reproductive efficiency in Nellore cattle is controlled by multiple loci acting on neuroendocrine signaling, energy metabolism, immune regulation, and development (Fig. 4a, Tables 1 and 2). These regions explained an important proportion of the additive genetic variance (see Additional file 2: Figs. S4a and S5a), supporting their relevance for baseline differences in reproductive performance across heifers.

Candidate genes within BTA1 (Tables 1 and 2) are related to cellular homeostasis, gametogenesis, neural development, and stress responses, thereby linking reproductive function and environmental adaptability, but functional validation in Nellore remains limited. On BTA3, a region explaining 0.95% (HP) and 1.35% (HR) of the genetic variance harbors *OLFML3*, *HIPK1*, and *PTPN22*. The *OLFML3* gene is associated with placental and embryonic development, and its dysregulation can lead to impaired pregnancy [76]. The *HIPK1* gene mediates stress-adaptive response and has been identified under positive selection in tropical breeds due to its role in oxidative stress resistance [77]. The *PTPN22* contributes to immune tolerance during early gestation, affecting fertility through uterine immune regulation [78]. On BTA22 (54.26–54.75), genes such as *ATP2B2* and *HRH1*, implicated in calcium signaling physiological responses essential for ovulation [79] and showed strong SNP × E effects (Figs. 5 and 6).

Regions associated with muscle development also showed strong SNP × E effects, particularly on BTA16 mapping the genes *ZBED6* (BTA16 1.45–1.7), *MYOG*, and *MYBPH* (BTA16 0.81–1.31) consistently associated with both traits (Figs. 5 and 6). The *ZBED6* gene represses growth-related genes such as *IGF2*, modulating lean tissue deposition and growth potential [80], whereas myogenic factors (*MYOG* and *MYBPH*) promote muscle differentiation and contractility, supporting a faster growth [81]. For HP, increased SNP effects under favorable EG (Fig. 5), supporting fast muscle development to reach an ideal body weight (~60.9% of mature weight) before 18 months [1, 82]. For HR, the same pattern under favorable EG (Fig. 6) indicates a role in postpartum recovery through better energy maintenance. In stressful tropical systems, these loci link somatic development to reproductive efficiency via insulin–glucose homeostasis and energy partitioning [83].

Reproductive performance in heifers is largely coordinated by neuroendocrine mechanisms that regulate puberty onset and gonadotropin secretion through the HPG axis [9, 84]. The GWAS and fine-mapping identified the genes *KISS1* (BTA16, 1.70–2.01 Mb) and *GnRHR* (BTA6, 83.15–83.49 Mb) as candidate genes for both HP and HR (see Additional file 2: Fig. S7). *KISS1* acts as a gatekeeper of puberty onset acting through stimulation of GnRH secretion and HPG axis activation [85], whereas *GNRHR* mediates GnRH signaling and regulates the secretion of luteinizing hormone (LH) and follicle-stimulating hormone (FSH) [86]. SNP effects in both regions increased under more favorable EG, indicating that better environments enhance the expression of genetic differences in neuroendocrine control of reproduction [9, 87].

The *GHRL* gene region on BTA22 (54.26–54.75 Mb) showed a strong SNP × E interaction for HP (Fig. 5) and HR (Fig. 6), indicating that genetic variation in this pathway may contribute to differences in reproductive performance across environments. Given the role of ghrelin in linking energy balance to reproductive function, this result indicates that variation in this pathway becomes more relevant when nutritional and management conditions are adequate to support growth, puberty onset, and reproductive activity [88]. The *GHRL* gene combined with the *KISS1* and *GNRHR* genes, connects energy status signals to reproductive function, adjusting physiological processes to align with environmental conditions and available energy resources.

Under lower EG levels, reproductive success depends on the ability of heifers to prioritize energy allocation among growth, maintenance, and reproduction [67]. In this context, GWAS and fine-mapping identified a relevant region on BTA5 (66.43–66.52 Mb) harboring *IGF1*, *PMCH*, and *PARPBP* (Additional file 2: Fig. S7), supporting the role of metabolic regulation and somatic development in both HP and HR across environments [8, 68]. *IGF1* is directly related to follicular development and GnRH–LH signaling in response to nutritional and environmental cues, affecting reproductive maturity and cyclicity [9, 89]. The *PMCH* gene is associated with energy balance, fat deposition, and feed intake via neuropeptides (*MCH, NEI,* and *NGE*), which may indirectly affect reproductive readiness [90–92]. In Nellore heifers, genetic variants in the *PMCH* gene have been associated with sexual precocity [9] and related metabolic processes [90], reinforcing the importance of this region in extensive production environments. On the other hand, *PARPBP* gene supports metabolism, ovarian follicle formation and cellular repair processes [93, 94]. These results indicate that variation in this BTA5 region contributes to differences in reproductive performance, particularly under conditions in which energy availability limits sexual development and postpartum recovery in heifers [90, 91, 95, 96].

Significant regions on BTA6 and BTA16 exhibited strong SNP×E effects for both HP (Fig. 5) and HR (Fig. 6) traits. These regions mapped the genes *ADORA1* (BTA16, 0.81–1.31 Mb), *ETNK2* (BTA16, 1.70–2.01 Mb), *PLEKHA6* (BTA16, 1.70–2.01 Mb), and *SULT1B1* (BTA6, 85.00–85.17 Mb), indicating that genes related to metabolic and cellular homeostasis contribute to environmental sensitivity in reproductive performance [97–99]. SNP effects in these regions increased as EG improved, showing that their contribution to HP and HR becomes more evident under more favorable conditions (Figs. 5 and 6). These results reinforce that metabolic homeostasis is a central component of genotype-by-environment differences in early reproductive performance [85].

SNPs near the *MAPK3* gene (BTA25 25.93–26.43 Mb) exhibited strong SNP×E effects on HP and HR (Figs. 5 and 6). The gene *MAPK3* encodes ERK1 a key component of the ERK/MAPK pathway involved in oocyte maturation, ovulation, and LH-dependent gene expression in the bovine ovulatory follicle, supporting this region as relevant to reproductive performance [73, 100]. In the same region, *SH2B1* showed strong environment-dependent effects on HP (Fig. 5) and HR (Fig. 6). *SH2B1* plays a key role in energy metabolism, thereby regulating insulin and leptin signaling, this result links energy homeostasis with puberty attainment and postpartum cyclicity [101, 102]. Indicating that under favorable EG suggests that genetic differences in energy-sensing pathways become more evident when environmental conditions support adequate growth and metabolic stability.

The *MTNR1A* gene (BTA27 16.22–16.61 Mb) encodes a melatonin receptor that mediates the effects of melatonin on the HPG axis and influences ovarian function, including reproductive cyclicity [103]. In females, variants in *MTNR1A* have been linked to puberty onset and reproductive efficiency, supporting its role as a candidate gene for fertility traits [104]. The identified genes for intercept parameters of RN illustrate how metabolic and endocrine regulation converge to support reproductive resilience to environmental disturbances (Figs. 5 and 6). Heifers carrying favorable alleles at these loci exhibit earlier puberty and shorter postpartum intervals, improving HP and HR under variable EGs (Fig. 3).

#### Genomic regions with effect on slope

Combined GWAS and fine-mapping analyses identified genomic regions across multiple chromosomes associated with the slope of HP and HR, indicating genotype-specific differences in environmental responsiveness (Figs. 5 and 6; Table 3). In general, SNP effects increased under more favorable EG, showing that genetic differences in reproductive efficiency become more evident as environmental constraints are reduced. The main regions harbored genes related to growth, insulin–glucose homeostasis, and endocrine regulation, which is consistent with the biological basis of reproductive performance [2, 8, 105].

The significant genomic regions exhibited high G×E effects comprising key components of the somatotropic axis and insulin-glucose signaling pathways essential for controlling reproductive functions [16, 106] and explained a substantial portion of the total additive genetic variance for HP (0.42% to 2.66%) and HR (0.46% to 2.38%). These clusters included regions on BTA2 (*IGFBP2* and *IGFBP5*), 7 (*INSR*), 29 (*INS* and *IGF2*), and 3 (*HSD17B7*), with higher SNP effects under favorable EG (Fig. 5 and 6). These loci are functionally related to biological mechanisms involved on the modulation of body composition and nutrient partitioning, which enable heifers to meet metabolic thresholds for earlier puberty [9] and improved rebreeding [107]. Notably, upregulation of *INSR*, *INS*, *IGF2,* and *HSD17B7* increases the availability of metabolic substrates, supporting oocyte maturation and overall fertility [108], while downregulation of *IGFBP2* and *IGFBP5* enhances IGF-1 and gonadotropin bioavailability, stimulating follicular development and estrous cyclicity [109]. Additionally, genes *ANGPTL4* (BTA7 16.96–17.23 Mb) and *MTHFD1* (BTA10 76.4–76.47 Mb) associated with energy balance and growth reinforce the importance of energy balance, lipid metabolism, and growth-related processes in the environmental sensitivity of HP and HR [110, 111].

Genomic regions linked to neuroendocrine and metabolic mechanisms, such as BTA13 (66.18–66.25 Mb) and BTA17 (41.11–41.98 Mb), showed strong SNP × E effects for HP and HR. On BTA13, variants near *GHRH* increased in effect as EG improved (Figs. 5 and 6), which is biologically consistent with the role of the GH–IGF axis in linking nutritional status, growth, and puberty [112, 113]. This indicates the role of *GHRH* in mediating adaptive reproductive responses under variable management conditions (Figs. 5 and 6). On BTA17 (41.11–41.98 Mb), SNPs mapping neurotransmission genes *GRIA2* and *GLRB* (Table 3) showed marked environment-dependent effects (Figs. 5 and 6). Biologically, the *GRIA2* gene encodes glutamate receptors that stimulate GnRH neuron activity, while the *GLRB* gene encodes glycine receptors that exert inhibitory effects [114, 115]. Under adequate EG levels, enhance neuroendocrine pathway which reduces the puberty onset and affects the postpartum estrous return [105]. On the other hand, low levels of EG weaken excitatory signals and increase inhibitory tone, delaying puberty and negatively impacting HR.

The significant region on BTA6 (64.93–65.28 and 65.45–65.91) surrounds the genes *GABRA1*, *GABRA2*, and *GABRA4,* which modulate GnRH neuron activity through GABAergic signaling, thereby influencing the HPG axis [116]. Increased GABAergic signaling suppresses GnRH release, reduces LH and FSH secretion, and impairs follicular development and ovulation [117, 118]. Nevertheless, lower GABRA expression has been associated with earlier puberty, greater follicular maturation, improved ovarian cyclicity [117, 118] and better HR after first calving through faster recovery from postpartum anestrus [119]. In this study, SNP effects near *GABRA* genes decreased as EG improved (Figs. 5 and 6), suggesting that genetic and environmental modulation of GABA pathways enhances sexual precocity, shortens postpartum anestrus, and improves reproductive efficiency in beef cattle.

A genomic hotspot on BTA11 (30.98–31.69 Mb), harboring *LHCGR* and *FSHR*, showed strong SNP × E effects for HP (Fig. 5) and HR (Fig. 6) as the EG improved (Table 3). These genes encode LH and FSH receptors, essential for follicular development and ovulation, and their allelic variation has been associated with fertility-related traits [120]. Under better nutritional and management conditions, favorable alleles at this region are likely to enhance gonadotropin responsiveness and support ovarian activity, improving HP and HR (Figs. 5 and 6).

Regulatory mechanisms at the ovarian level were identified by SNP × E interactions in genomic regions containing steroid and growth factor receptor genes (Table 3). The *ESR2* gene, located on BTA10 (76.4–76.47 Mb)*,* encodes estrogen receptor β (Table 3) and exhibited clear environment-dependent effects on HP (Fig. 5) and HR (Fig. 6), varying from a negative SNP effect (EG = −3.0) to positive effect (EG = 3.0) EG levels. These results suggest that favorable *ESR2* alleles enhance estrogen-mediated feedback, promoting follicular maturation and improving reproductive efficiency under adequate conditions (EG > 0), while nutritional stress (EG < 0) reduces estrogen synthesis, limiting *ESR2* signaling and reducing reproductive efficiency [105].

*EGFR* on BTA22 (0.81–0.96 Mb, Table 3) exhibited environment-dependent effects on HP (Fig. 5) and HR (Fig. 6), with negative effects under low EG and positive effects under high EG. *EGFR* mediates EGF signaling, which is crucial for oocyte maturation, and ovulatory response through LH receptor regulation [121, 122]. The results suggest that low EG levels downregulate these pathways, impairing reproductive performance, whereas higher EG activates EGFR-mediated mechanisms, favoring puberty onset and the resumption of cyclicity [123]. Together, these results indicate that genes controlling ovarian signaling (*LHCGR*, *FSHR*, *ESR2* and *EGFR*) are highly sensitive to the environment. Their effects on HP and HR become pronounced only when endocrine conditions allow full expression. These interactions highlight the need to align genetic selection with optimal management to accelerate reproductive efficiency in beef heifers.

#### Genomic regions with shared effect on intercept and slope

We identified genomic regions on BTA4 (92.38–93.81 Mb) and BTA14 (23.03–24.06 Mb) with pleiotropic effects on both genetic potential (intercept) and environmental responsiveness (slope) for reproductive efficiency traits (HP and HR). This indicates shared genetic control of baseline performance and sensitivity to the environment. In both regions, SNP effects were higher under favorable EG (Figs. 5 and 6), suggesting that their influence on energy balance, metabolism, and reproductive signaling becomes more evident under better conditions.

The BTA4 region (92.28–93.38 Mb and 93.75–93.81 Mb), encompasses key regulators involved in the integration of metabolic status and reproductive function, *LEP*, *HILPDA*, *SND1* and *IRF5* (Tables 1 and 3). These loci modulate the HPG axis in response to energetic and environmental conditions [105, 124]. Among them, the gene *LEP* plays a key role in energy homeostasis and reproductive axis activation, signaling body energy reserves to the hypothalamus and contributing to puberty onset and maintenance of reproductive function [125]​. The higher SNP effects observed under favorable EG suggest that LEP acts as a metabolic gatekeeper only when nutritional requirements are met [67]. Complementing this axis, *HILPDA* may reinforce this effect by regulating lipid droplet formation and lipolysis through inhibition of adipose triglyceride lipase [126], helping maintain energy availability for reproduction [9, 127]. These genes support a metabolic–reproductive interface that can improve HP and HR, especially under nutritional or climatic stress.

The *SND1* gene which is associated with RNA processing and cholesterol metabolism, showed increasing SNP effects under high EG (Figs. 5 and 6), consistent with a greater contribution to steroidogenesis when cholesterol is no longer limiting [128]. Under energy restriction, reproductive suppression may mask this effect, whereas under favorable conditions its transcriptional and metabolic roles may be fully expressed [129]. The *IRF5* regulates key pro-inflammatory cytokines (IL-1β, TNF-α, IL-6, and IL-1α) essential for ovarian function [130], but excessive inflammation impairs fertility [131]. Under stressful conditions, alleles associated with tighter inflammatory control may help preserve ovarian function by reducing damage related to lipolysis and oxidative stress [131], thereby increasing the probability of HP and HR.

The pleiotropic region identified by GWAS and fine-mapped on BTA14 harbors the genes *MOS, PLAG1, LYN, PENK,* and *CHCHD7*, which are involved in puberty, growth, and hormone regulation [21, 132–135]. Specifically, the gene *PLAG1* in this region affects the IGF1/IGF2 axis and HPG regulation [136], establishing a functional link between somatic growth and reproductive efficiency. Variation in SNP effects across environments (Figs. 5 and 6) indicate that differences in reproductive efficiency are mainly driven by growth-related endocrine pathways, particularly IGF signaling [89, 137]. In addition, the *PENK* gene plays a biological role in response to stress and homeostasis [138], encoding an opioid precursor essential for neuronal activation, highlighting the role of opioid signaling in the neuroendocrine regulation of puberty onset [139]. Likewise, *MOS* acts through the MOS/MAPK cascade, which is essential for oocyte maturation and disruption of this pathway has been associated with reduced fertility [140]. These findings indicate that genetic variation in this region may affect fertility through mechanisms related to oocyte maturation and early embryonic development, potentially interacting with pathways involved in energy metabolism, prostaglandin synthesis, and lipid homeostasis [134].

These results show that reproductive traits in Nellore heifers are controlled by genomic regions linked to metabolic, neuroendocrine, and inflammatory pathways. The SNP effects vary with the environment, indicating that reproductive performance depends not only on genetic makeup but also on nutritional and climate conditions. This context-dependent expression highlights the need to account for environmental sensitivity in breeding programs targeting reproductive resilience in tropical systems.

### Genetic control of reproductive potential (intercept) through metabolic and neuroendocrine integration

Biological pathways associated with the intercept of the RN for HP and HR traits uncover a tightly regulated system that connects energy sensing, hypothalamic activation, gonadal function, and stress response (Table 6). These pathways appear to shape the genetic baseline of reproductive potential captured by the intercept, and their functional integration offers a molecular basis for reproductive efficiency in beef cattle under tropical production conditions.

The identified Leptin signaling pathway (R-BTA-2586552), driven by *LEP* and *SH2B1*, regulates energy balance and supports activation of the reproductive axis. In this context, leptin acts as a permissive of energy sufficiency, relaying metabolic status to hypothalamic circuits upstream of *KISS1* and *GnRH*, at least in part through the JAK–STAT pathway (bta04630) [141]. Additionally, the enrichment of the ghrelin pathway (R-BTA-422085), which includes the *GHRL*, *LEP*, and *IGF1* genes, highlights a finely balanced system in which anabolic and catabolic signals jointly tune the reproductive axis. Under nutrient restrictions, ghrelin suppresses gonadotrophin pulsatility and compromises follicular function, providing a plausible mechanism by which metabolic stress delays puberty and postpartum recovery [71]. These results suggest that genetic variation in these pathways contributes to the genetic potential for reproductive capacity captured by the intercept, particularly under varying nutritional conditions.

These metabolic signals converge on the G alpha (q) signaling pathway (R-BTA-416476), by the genes *KISS1*, *GNRHR*, *PMCH*, and *HRH1* within a neuroendocrine module that links hypothalamic input to ovarian function. Consistent with the canonical role of GnRH receptor signaling, this pathway couples calcium mobilization and PKC activation to pulsatile gonadotrophin release, thereby connecting central sensing of metabolic state to follicular maturation and luteal function [142]. Its overlap with GnRH signaling (bta04912) and progesterone-mediated oocyte maturation (bta04914) supports a coordinated framework for sexual precocity and successful rebreeding [143].

Within this regulatory architecture, *MAPK3* (ERK1) emerges as a signaling hub linking GnRH signaling (bta04912), MAPK cascade (bta04010), growth hormone signaling (bta04935), oocyte maturation, and the heat shock response. This is consistent with the established role of ERK1/2 signaling in ovulation, granulosa-cell differentiation and luteinization [144]. Its functional enrichment within the HSF1-mediated heat-shock pathway (R-BTA-3371453) further supports a link between thermal stress and reduced reproductive performance, as heat stress impairs follicular dynamics and oocyte competence in cattle [145]. The JAK–STAT pathway (bta04630) adds a complementary layer by linking metabolic and cytokine signaling, whereas the *LEP*, *IL27* and *CTF1* genes point to mechanisms potentially relevant to uterine function and post-partum recovery [146]. Enrichment of the glucose-metabolism pathway (R-BTA-70326) likewise supports the view that the RN intercept captures a baseline integrating metabolic, neuroendocrine and stress-response processes that are all relevant to female fertility [15].

These results support a regulatory network in which neuroendocrine (*LEP*, *GHRL* and *KISS1*), metabolic (*IGF1* and *ALDOA*), and stress-related (*MAPK3* and *HSF1*) genes interact to shape the genetic baseline (intercept) of fertility under challenging conditions. Rather than acting independently, these pathways likely operate as an integrated system, such that variation at one level may influence the overall reproductive response. Animals carrying favorable alleles across this network may therefore be better able to maintain reproductive function across heterogeneous environments. Because many of these genes participate in environmentally responsive pathways, their effects are also plausible substrates for G × E, potentially contributing to sire reranking across production conditions. In this context, the RN intercept captures not only baseline reproductive potential, but also part of the biological architecture underlying resilience in tropical beef systems.

### Genetic control of reproductive responsiveness (slope) through reproductive pathways

The reproductive response of Nellore heifers to environmental improvement appears to be shaped by the plasticity of molecular pathways that integrate metabolic and neuroendocrine signals. Animals with high slopes for HP and HR are therefore expected to respond more readily to favorable conditions, activating pathways that initiate, sustain and coordinate reproductive function.

Pathways related to ovarian steroidogenesis (bta04913), estrogen signaling (bta04915) and prolactin signaling (bta04917) were significantly enriched, consistent with greater responsiveness of the hypothalamic–pituitary–gonadal axis in high-slope animals (Table 6). Under improved energy balance, increases in insulin and IGF1 can enhance steroidogenesis in granulosa and theca cells, particularly estradiol production, which is required for the pubertal LH surge and for the resumption of postpartum cyclicity [147]. Variants associated with the RN slope may therefore mark genotypes that more efficiently upregulate steroidogenic enzymes and estrogen receptors, coupling endocrine feedback to metabolic status and aligning growth with reproductive readiness [148]. Prolactin signaling may add a complementary layer to this response. Although excessive prolactin suppresses GnRH pulsatility, moderate signaling is associated with oocyte competence, luteal maintenance, and early embryo development [149]. High-slope genotypes may thus benefit from prolactin-dependent reproductive functions without compromising GnRH secretion, particularly when nutritional conditions improve.

Significant metabolic pathways were also enriched, including insulin receptor signaling, AMPK/MAPK signaling, adipogenesis, hormone signaling and GABAergic synapse pathways (Table 6; *P*-value < 0.05), pointing to coordinated regulation of reproductive responsiveness across the environmental gradient [8, 16]. Enrichment of insulin signaling (R-BTA-74752) and MAPK signaling (bta04010), involving *INS, INSR, IGF2, DUSP8* and *EGFR*, is consistent with roles in growth and cellular metabolism as well as in hypothalamic–pituitary control of reproductive timing [150]. This is particularly relevant in tropical beef systems, in which heifers are exposed to seasonal variation in nutrition and heat load. These findings are also consistent with previous studies linking insulin signaling to puberty onset and ovarian responsiveness in cattle [151].

The adipogenesis pathway (R-BTA-9843745), represented by the *LEP*, *ANGPTL4* and *ZNF423* genes, further highlights the importance of body energy reserves in reproductive readiness. Leptin acts as a permissive metabolic signal for activation of the hypothalamic–pituitary–gonadal axis [16] and variation in *LEP* expression or sensitivity has been associated with differences in age at puberty and rebreeding success under nutritional restriction [83]. *ZNF423* is of particular interest because it links metabolic and ovarian function. In the ovary, it regulates estrogen-responsive genes and promotes granulosa-cell differentiation, both of which are required for folliculogenesis and fertility [152]. Simultaneously, it controls preadipocyte commitment and lipid accumulation, with epigenetic activation promoting differentiation into mature adipocytes [153].

Enrichment of the GABAergic synapse pathway (bta04727), involving *GABRA2*, *GABRA4*, *GABRB1*, *ADCY7* and *SRC*, further suggests that central nervous system mechanisms contribute to variation in responsiveness to environmental change. Because GABAergic neurons regulate pulsatile GnRH release [116], genetic variation in this pathway may influence how effectively animals adjust reproductive function to environmental conditions. Its overlap with estrogen (bta04915) and prolactin (bta04917) signaling, through genes such as *FSHR*, *LHCGR*, *ESR2* and *ADCY7*, further supports a role for receptor-mediated signaling in puberty, follicular development and ovulation [154].

In summary, variation in environmental conditions is associated with concerted physiological and molecular responses encompassing metabolic sensing, neuroendocrine signaling, and gonadal function. Heifers with steeper reaction norm slopes exhibit enhanced coordination among these pathways, effectively translating improvements in environmental conditions into reproductive activation. This integration is mediated by genetic networks that regulate hormonal signaling, intracellular transduction, and neuroendocrine homeostasis. Such a framework provides a mechanistic basis for the phenotypic plasticity observed in key reproductive traits and identifies biological processes with potential utility as targets for genomic selection and management in tropical beef production systems.

## Conclusions

Our results reveal a pronounced impact of genotype-by-environment (G × E) interactions on reproductive performance in Nellore heifers, mediated by genomic regions that influence both genetic potential and environmental sensitivity. Genomic breeding values for heifer pregnancy (HP) and rebreeding (HR) were significantly influenced by environmental variation, with stronger G × E interactions and lower sire rank coincidence observed under more divergent environmental conditions. This was particularly evident in environments with high variability, suggesting that nutritional and management factors modulate the expression of genetic merit for fertility and reproductive performance.

Key genomic regions controlling both baseline reproductive efficiency (intercept) and environmental responsiveness (slope) were identified. Genomic loci associated with intercept variability involved genes associated with fundamental processes such as lipid storage, embryonic development, and neuroendocrine regulation (e.g., *IGF1*, *KISS1,* and *GNRHR*). Conversely, the slope was predominantly influenced by loci related to adaptive metabolic and endocrine pathways, notably the insulin–IGF axis (*INS, IGF2,* and *IGFBP2/5*), gonadotropic receptors (*FSHR*, *LHCGR*), and GABAergic signaling (*GABRA2* and *GABRB1*), critical for reproductive plasticity and adaptability under environmental variation. Notably, genomic regions on BTA4 and BTA14 emerged as pleiotropic control for intercept and slope, encompassing genes crucial for energy homeostasis, metabolic regulation, and reproductive signaling (*LEP*, *PLAG1*, *HILPDA*, *SND1* and *MOS*). The combined effect of these loci underscores their central role in managing reproductive performance and environmental resilience. Integrating these genomic insights into breeding strategies will enable precise selection of animals with both high reproductive potential and robust adaptive responses, ultimately enhancing reproductive outcomes, genetic progress, and sustainability in tropical and subtropical beef production systems.

## Supplementary Information

Additional file 1: Table S1. Number of SNP markers, first and last marker positions, chromosome length, and number of independent segmentsper chromosome.

Additional file 2: Fig. S1 Farm-level incidence of heifer early pregnancy at 16 months and heifer rebreeding after first calving. (a) Incidence of heifer early pregnancy at 16 months (HP) across farms, showing the proportion of precocious and non-precocious heifers in each herd. (b) Incidence of heifer rebreeding (HR) across farms, showing the proportion of rebreed and non-rebreed heifers in each herd. Fig. S2 Principal component analysisbased on the genomic relationship matrixof the individuals included in the genotype imputation framework, considering the first two principal components. Each point represents an individual animal, colored according to its role in the imputation process. Reference (red) and Target (green). The reference population comprises individuals genotyped at high densityand (HD) used as the reference panel, whereas the target population comprises individuals genotyped at low to medium density, whose genotypes were imputed to HD. PC1 and PC2 explain 22.4% and 12.4% of the total genetic variation, respectively. The substantial overlap between the reference and target populations indicates strong genetic representativeness between panels, a key prerequisite for accurate imputation from low–medium density to HD in structured breeding populations. Fig. S3 Principal component analysis based on genome-wide genotype data for all individuals included in the imputation pipeline from high-densitygenotypes (HD) to whole-genome sequence resolution (WGS). PC1 and PC2 explain 15.67% and 8.69% of the total genetic variance, respectively. Points represent individuals and are colored by their role in WGS imputation: HD-genotyped animals and HD-genotyped sires, which form the target set to be imputed to WGS, and WGS-sequenced sires, which constitute the reference panel. The strong overlap between target and reference groups across the first two PCs indicates that the WGS reference panel is genetically representative of the HD-imputed individuals, a key condition for robust imputation from HD to WGS. Fig. S4 Manhattan plots showing SNP-based partitioning of additive genetic variance for heifer early pregnancy (HP), analyzed under reaction norm model parameters intercept and slope. These results highlight distinct genomic regions contributing to the genetic potentialand environmental sensitivityof HP. Fig. S5 Manhattan plots showing SNP-based partitioning of additive genetic variance for heifer rebreeding (HR), analyzed under reaction norm model parameters intercept and slope. These results highlight distinct genomic regions contributing to the genetic potentialand environmental sensitivityof HR. Fig. S6 Linkage disequilibrium (LD) heatmaps of genomic regions significantly associated with the intercept parameter of the reaction norm model for heifer early pregnancy (HP) and heifer rebreeding (HR) traits, identified via multi-trait GWAS statistical combination. The heatmaps depict r^2^-based pairwise LD between SNPs within each significant region in the HD SNP chip. Each panel shows the chromosome number, mean LD, and physical length of the region. Fig. S7 Fine-mapping of genomic regions significantly associated with the intercept parameter of the reaction norm model for heifer early pregnancy (HP; a) and heifer rebreeding (HR; b). Manhattan plots based on posterior inclusion probabilities (PIP) estimated by FINEMAP using imputed WGS data (±100 kb from lead SNPs). Peaks with PIP > 0.90 and log_10_(BF) > 3.5 suggest potential causal roles in determining the genetic merit for reproductive traits. Results refer to significant regions previously detected in the multi-trait GWAS statistical combination. Fig. S8 Linkage disequilibrium (LD) heatmaps of genomic regions significantly associated with the slope parameter of the reaction norm model for heifer early pregnancy (HP) and heifer rebreeding (HR) traits, identified via multi-trait GWAS statistical combination. The heatmaps depict r²-based pairwise LD between SNPs within each significant region in the HD SNP chip. Each panel shows the chromosome number, mean LD, and physical length (in kb) of the region. Fig. S9 Fine-mapping of genomic regions significantly associated with the slope parameter of the reaction norm model for heifer early pregnancy (HP; a) and heifer rebreeding (HR; b). Manhattan plots showing the PIP estimates from FINEMAP within ±100 kb of lead SNPs associated with environmental responsiveness. Peaks with PIP > 0.90 and log_10_(BF) > 3.5 indicate variants with a strong probability of modulating environmental responsiveness for reproductive traits. These results refine the loci identified in the meta-GWAS analysis, pinpointing potential regulatory variants influencing plasticity. 

## Data Availability

No datasets were generated or analysed during the current study.

## Abbreviations

AI: Artificial insemination
BLUE: Best Linear Unbiased Estimate
BOA: Bayesian output analysis
BTA: *Bos taurus* autosome
CG: Contemporary group
GE: Environmental gradient
CNS: Central nervous system
EG: Environmental gradient
FDR: False discovery rate
GBLUP: Genomic Best Linear Unbiased Prediction
GEBV: Genomic estimated breeding value
GO: Gene Ontology
GWAS: Genome-wide association study
G × E: Genotype-by-environment interaction
HP: Heifer early pregnancy
HPG: Hypothalamic–pituitary–gonadal axis
HR: Heifer rebreeding
LD: Linkage disequilibrium
L: Genome length
LH: Luteinizing hormone
MAF: Minor allele frequency
ME: Number of independent chromosomal segments
NE: Effective population size
PCA: Principal component analysis
PIP: Posterior inclusion probability
PKC: Protein kinase C
QC: Quality control
RN: Reaction norm
*r*_g_: Genetic correlation
*r*: Pearson correlation between GEBV
SNP: Single nucleotide polymorphism
SNP×E: SNP-by-environment interaction
ssGBLUP: Single-step Genomic Best Linear Unbiased Prediction
ssGRN: Single-step genomic reaction norm
WGS: Whole-genome sequence
YW: Yearling weight
