# Supplementary material for: Multi-trait GWAS of reaction norm parameters reveals environment-responsive loci influencing reproductive performance in heifers
Source: J Anim Sci Biotechnol. 2026 Jun 8;17:113. doi: 10.1186/s40104-026-01428-5 (PMC13244917; doi:10.1186/s40104-026-01428-5)
Supplement: Supplementary file 1 — Additional file 1: Table S1. Number of SNP markers, first and last marker positions, chromosome length, and number of independent segmentsper chromosome. [file 40104_2026_1428_MOESM1_ESM.docx]

**Supplemental Information**

Table S1. Number of SNP markers, first and last marker positions, chromosome length (M), and number of independent segments (Me) per chromosome

| **Chromosome** | n. SNP | Start (bp) | End (bp) | Chromosome length (M) | Independent segments (Me) |
| --- | --- | --- | --- | --- | --- |
| 1 | 25316 | 790905 | 158493439 | 1.58 | 62.32 |
| 2 | 21920 | 83872 | 136128478 | 1.36 | 55.38 |
| 3 | 20419 | 160183 | 120951023 | 1.21 | 50.39 |
| 4 | 18565 | 182210 | 119798205 | 1.20 | 50.00 |
| 5 | 18263 | 32329 | 119970872 | 1.20 | 50.11 |
| 6 | 22123 | 143739 | 117767753 | 1.18 | 49.34 |
| 7 | 18389 | 130947 | 110030510 | 1.10 | 46.77 |
| 8 | 20181 | 110133 | 111435210 | 1.11 | 47.25 |
| 9 | 18548 | 64186 | 104550917 | 1.04 | 44.95 |
| 10 | 16150 | 61312 | 103158796 | 1.03 | 44.48 |
| 11 | 16790 | 92081 | 106942315 | 1.07 | 45.75 |
| 12 | 13532 | 90415 | 87206875 | 0.87 | 39.00 |
| 13 | 13700 | 460596 | 83457015 | 0.83 | 37.57 |
| 14 | 15426 | 162508 | 82379665 | 0.82 | 37.29 |
| 15 | 13557 | 95457 | 85005607 | 0.85 | 38.23 |
| 16 | 13488 | 228391 | 80909778 | 0.81 | 36.75 |
| 17 | 12280 | 59867 | 73127137 | 0.73 | 34.05 |
| 18 | 10959 | 199888 | 65811001 | 0.66 | 31.36 |
| 19 | 9388 | 54106 | 63432318 | 0.63 | 30.55 |
| 20 | 11742 | 193030 | 71856566 | 0.72 | 33.55 |
| 21 | 11738 | 289502 | 69838119 | 0.70 | 32.79 |
| 22 | 10048 | 159844 | 60703550 | 0.61 | 29.51 |
| 23 | 8865 | 26021 | 52407352 | 0.52 | 26.46 |
| 24 | 10395 | 377361 | 62231274 | 0.62 | 29.99 |
| 25 | 7005 | 179006 | 42212547 | 0.42 | 22.49 |
| 26 | 8828 | 202644 | 51973129 | 0.52 | 26.23 |
| 27 | 7536 | 1153324 | 45479594 | 0.44 | 23.38 |
| 28 | 6960 | 500625 | 45818142 | 0.45 | 23.77 |
| 29 | 7506 | 420542 | 51072873 | 0.51 | 25.81 |
