# Supplementary material for: Multi-trait GWAS of reaction norm parameters reveals environment-responsive loci influencing reproductive performance in heifers
Source: J Anim Sci Biotechnol. 2026 Jun 8;17:113. doi: 10.1186/s40104-026-01428-5 (PMC13244917; doi:10.1186/s40104-026-01428-5)
Supplement: Supplementary file 2 — Additional file 2: Fig. S1 Farm-level incidence of heifer early pregnancy at 16 months and heifer rebreeding after first calving. (a) Incidence of heifer early pregnancy at 16 months (HP) across farms, showing the proportion of precocious and non-precocious heifers in each herd. (b) Incidence of heifer rebreeding (HR) across farms, showing the proportion of rebreed and non-rebreed heifers in each herd. Fig. S2 Principal component analysisbased on the genomic relationship matrixof the individuals included in the genotype imputation framework, considering the first two principal components. Each point represents an individual animal, colored according to its role in the imputation process. Reference (red) and Target (green). The reference population comprises individuals genotyped at high densityand (HD) used as the reference panel, whereas the target population comprises individuals genotyped at low to medium density, whose genotypes were imputed to HD. PC1 and PC2 explain 22.4% and 12.4% of the total genetic variation, respectively. The substantial overlap between the reference and target populations indicates strong genetic representativeness between panels, a key prerequisite for accurate imputation from low–medium density to HD in structured breeding populations. Fig. S3 Principal component analysis based on genome-wide genotype data for all individuals included in the imputation pipeline from high-densitygenotypes (HD) to whole-genome sequence resolution (WGS). PC1 and PC2 explain 15.67% and 8.69% of the total genetic variance, respectively. Points represent individuals and are colored by their role in WGS imputation: HD-genotyped animals and HD-genotyped sires, which form the target set to be imputed to WGS, and WGS-sequenced sires, which constitute the reference panel. The strong overlap between target and reference groups across the first two PCs indicates that the WGS reference panel is genetically representative of the HD-imputed individuals, a key conditio [file 40104_2026_1428_MOESM2_ESM.docx]

# Supplemental Information

Figure S1. Farm-level incidence of heifer early pregnancy at 16 months and heifer rebreeding after first calving. (a) Incidence of heifer early pregnancy at 16 months (HP) across farms, showing the proportion of precocious and non-precocious heifers in each herd. (b) Incidence of heifer rebreeding (HR) across farms, showing the proportion of rebreed and non-rebreed heifers in each herd.

Figure S2. Principal component analysis (PCA) based on the genomic relationship matrix (GRM) of the individuals included in the genotype imputation framework, considering the first two principal components. Each point represents an individual animal, colored according to its role in the imputation process. Reference (red) and Target (green). The Reference population comprises individuals genotyped at high density (HD) and used as the reference panel, whereas the Target population comprises individuals genotyped at low to medium density, whose genotypes were imputed to HD. PC1 and PC2 explain 22.4% and 12.4% of the total genetic variation, respectively. The substantial overlap between the Reference and Target populations indicates strong genetic representativeness between panels, a key prerequisite for accurate imputation from low–medium density to HD in structured breeding populations.

Figure S3. Principal component analysis (PCA) based on genome-wide genotype data for all individuals included in the imputation pipeline from high-density (HD) genotypes to whole-genome sequence (WGS) resolution. PC1 and PC2 explain 15.67% and 8.69% of the total genetic variance, respectively. Points represent individuals and are colored by their role in WGS imputation: HD-genotyped animals (red) and HD-genotyped sires (blue), which form the Target set to be imputed to WGS, and WGS-sequenced sires (gray), which constitute the Reference panel. The strong overlap between Target and Reference groups across the first two PCs indicates that the WGS reference panel is genetically representative of the HD-imputed individuals, a key condition for robust imputation from HD to WGS.

Figure S4. Manhattan plots showing SNP-based partitioning of additive genetic variance for heifer early pregnancy (HP), analyzed under reaction norm model parameters (a) intercept and (b) slope. These results highlight distinct genomic regions contributing to the genetic potential (intercept) and environmental sensitivity (slope) of HP.

Figure S5. Manhattan plots showing SNP-based partitioning of additive genetic variance for heifer rebreeding (HR), analyzed under reaction norm model parameters (a) intercept and (b) slope. These results highlight distinct genomic regions contributing to the genetic potential (intercept) and environmental sensitivity (slope) of HR.

Figure S6. Linkage disequilibrium (LD) heatmaps of genomic regions significantly associated with the intercept parameter of the reaction norm model for heifer early pregnancy (HP) and heifer rebreeding (HR) traits, identified via multi-trait GWAS statistical combination. The heatmaps depict r²-based pairwise LD between SNPs within each significant region in the HD SNP chip. Each panel shows the chromosome number, mean LD, and physical length (in kb) of the region.

Figure S7. Fine-mapping of genomic regions significantly associated with the intercept parameter of the reaction norm model for heifer early pregnancy (HP - a) and heifer rebreeding (HR - b). Manhattan plots based on posterior inclusion probabilities (PIP) estimated by FINEMAP using imputed WGS data (±100 kb from lead SNPs). Peaks with PIP > 0.90 and log_10_(BF) > 3.5 suggest potential causal roles in determining the genetic merit for reproductive traits. Results refer to significant regions previously detected in the multi-trait GWAS statistical combination.

Figure S8. Linkage disequilibrium (LD) heatmaps of genomic regions significantly associated with the slope parameter of the reaction norm model for heifer early pregnancy (HP) and heifer rebreeding (HR) traits, identified via multi-trait GWAS statistical combination. The heatmaps depict r²-based pairwise LD between SNPs within each significant region in the HD SNP chip. Each panel shows the chromosome number, mean LD, and physical length (in kb) of the region.

Figure S9. Fine-mapping of genomic regions significantly associated with the slope parameter of the reaction norm model for heifer early pregnancy (HP - a) and heifer rebreeding (HR - b). Manhattan plots showing the PIP estimates from FINEMAP within ±100 kb of lead SNPs associated with environmental responsiveness. Peaks with PIP > 0.90 and log_10_(BF) > 3.5 indicate variants with a strong probability of modulating environmental responsiveness for reproductive traits. These results refine the loci identified in the meta-GWAS analysis, pinpointing potential regulatory variants influencing plasticity.
